# Workforce diversity in specialist physicians: Implications of findings for religious affiliation in Anaesthesia & Intensive Care

**DOI:** 10.1371/journal.pone.0288516

**Published:** 2023-08-23

**Authors:** Ali Alim-Marvasti, Mohammed Jawad, Chibueze Ogbonnaya, Ali Naghieh

**Affiliations:** 1 UCL Queen Square Institute of Neurology, University College London, London, United Kingdom; 2 Imperial College Healthcare NHS Trust, London, United Kingdom; 3 Institute of Child Health, University College London, London, United Kingdom; 4 School of Public Policy, University College London, London, United Kingdom; 5 Middlesex University Business School, London, United Kingdom; University of Sharjah College of Health Sciences, UNITED ARAB EMIRATES

## Abstract

**Background:**

Minority ethnic identification between physician and patient can reduce communication and access barriers, improve physician-patient relationship, trust, and health outcomes. Religion influences health beliefs, behaviours, treatment decisions, and outcomes. Ethically contentious dilemmas in treatment decisions are often entangled with religious beliefs. They feature more in medical specialties such as Anaesthesia & Intensive Care, with issues including informed consent for surgery, organ donation, transplant, transfusion, and end-of-life decisions.

**Methods:**

We investigate diversity in religious affiliation in the UK medical workforce, using data from the General Medical Council (GMC) specialist register and Health Education England (HEE) trainee applications to medical specialties. We performed conservative Bonferroni corrections for multiple comparisons using Chi-squared tests, as well as normalised mutual-information scores. Robust associations that persisted on all sensitivity analyses are reported, investigating whether ethnicity or foreign primary medical qualification could explain the underlying association.

**Findings:**

The only significant and robust association in both GMC and HEE datasets affecting the same religious group and specialty was disproportionately fewer Anaesthesia & Intensive Care physicians with a religious affiliation of “Muslim”, both as consultants (RR 0.57[0.47,0.7]) and trainee applicants (RR 0.27[0.19,0.38]. Associations were not explained by ethnicity or foreign training. We discuss the myriad of implications of the findings for multi-cultural societies.

**Conclusions:**

Lack of physician workforce diversity has far-reaching consequences, especially for specialties such as Anaesthesia and Intensive Care, where ethically contentious decisions could have a big impact. Religious beliefs and practices, or lack thereof, may have unmeasured influences on clinical decisions and on whether patients identify with physicians, which in turn can affect health outcomes. Examining an influencing variable such as religion in healthcare decisions should be prioritised, especially considering findings from the clinician-patient concordance literature. It is important to further explore potential historical and socio-cultural barriers to entry of training medics into under-represented specialties, such as Anaesthesia and Intensive Care.

## 1. Introduction

Extensive research has examined the effects of ethnicity and minority identification on healthcare and health disparities [1, 2]. Ethnicity is defined as a person’s cultural identity, which includes language, customs and religion [3, 4]. Differences in culture, beliefs and communication issues affect access to services, service uptake and healthcare outcomes [5–8]. An expanding body of literature has reported significant influence of culture and religion on health beliefs, health choices and treatment decisions [9–13]. The literature on cultural competency has stressed the practical challenges in understanding and effectively addressing ethnic, cultural and religious differences by healthcare systems and professionals in order to improve services and reduce health inequalities [14–17].

Building on the literature on physician-patient communication, various studies have examined ‘race concordant/discordant’ relationships, referring to whether the clinician and patient are of the same ethnicity or race [7, 18–20]. Although ‘race’ and ‘ethnicity’ are distinct anthropological concepts, this body of literature tends to use the terms interchangeably, which is quite prevalent in the medical literature.[3] Race concordant consultations are longer, and both patients and doctors express higher satisfaction levels which in turn increase patient adherence to treatment plans [21–23]. A review of the literature in medical communication found that physicians behave less affectively when interacting with ethnic minority patients, and those patients are in turn less affective, less verbally expressive, and unassertive [24]. Key predictors for these culture related communication problems from the literature include cultural differences in explanatory models of health and illness, differences in cultural values, cultural differences in doctor-patient relationships, cultural bias, racism, and linguistic barriers. A study on pain management in minority groups found that cultural concordance in physician-patient dyads reduced self-reported and physiological indicators of pain in some minority groups [25]. Investigating the consequences of cultural discordance in clinician-patient relationships, Saha and Sanders [26] found that greater patient-perceived cultural distance from the treating clinician was associated with lower ratings of trust and perceived quality of care. To better understand this phenomenon, the authors conclude with the need to explore under-researched dimensions of culture, including biomedical and non-biomedical orientations towards health, and the role of religious/spiritual beliefs.

The literature thus points to the significance of physician workforce diversity and representation of ethnic minority groups in various medical specialties, with the potential for improving health outcomes and reducing health disparities [20]. From the ‘protected characteristics’ that represent diversity, as outlined by the UK Equalities Act 2010 and the US Civil Rights Act 1964, the range of literature that examine the link between minority groups and health outcomes are predominantly focused on race. Another protected characteristic that has significant influence on culture, health beliefs, treatment decisions, and health outcomes, is religion, which, as evident from the extant literature, is under-researched.

Religion and faith are an important component of ethnicity and influence people’s values, beliefs (including health beliefs), and behaviours. Many epidemiological studies have reported statistically significant beneficial effects of religious indicators on morbidity and mortality [27–30], which include health promoting behaviours originating from prescriptions governing alcohol, tobacco, general hygiene, and positive psychosocial influences [31, 32]. Religious organisations have contributed to healthcare by funding and supporting hospitals for vulnerable and underserved populations [33]. However, a body of literature has also explored the negative influences of religion on health. Asser and Swan [34] report negative outcomes and death in children resulting from parents’ reliance on faith-healing in place of medical treatment. In a longitudinal study, Pargament and Koenig [35] found that in elderly patients, an illness accompanied with religious distress (feeling punished by God or angry towards God, or feeling the devil was at work) was associated with a higher risk of mortality. Rosenbaum [36] discusses objections to contraception on religious grounds, and Swan [37] analyses a range of religious beliefs against medical treatments, including blood transfusions, open heart surgery, immunizations, prevention, and screening procedures. D’Souza [38] asserts that although clinicians should be objective, keeping their religious beliefs separate from their own practice, by doing so, they should not stray into dissociating patients’ beliefs and spiritual needs from their care. In considering the spiritual dimension of the patient, the clinician sends an important message that he or she is concerned with the person as a whole, enhancing the clinician-patient relationship and increasing the uptake and therapeutic impact of proposed interventions. Choudry, Latif [39] stresses the importance of this holistic perspective for palliative care clinicians, and the need for a deep understanding of the sociocultural and religious traditions observed by patient’s community. Considering the wide-ranging impact of religious affiliation on healthcare, Swihart, Yarrarapu [40] extensively discuss the need for ‘cultural religious competence’ for improving physician-patient relationships and subsequently health outcomes.

Medical ethics, and its interaction with religion, are increasingly relevant and topical across medical specialties [41–43]. However, some specialties may deal with ethically contentious dilemmas more frequently. These include Anaesthesia & Intensive Care (due to informed consent for surgery, decisions on organ donation, transplant, transfusion and end of life ceilings of treatment); Palliative Care (with decisions on end-of-life care); Psychiatry (due to disparate societal beliefs on the nature of mental health issues), and Obstetrics and Gynaecology (with contentions arising from termination of pregnancy and contraception).

Anecdotal reports by trainees suggested that there were fewer than expected Muslim doctors in Anaesthesia and Intensive Care in the UK. We therefore investigated the possibility of hitherto unknown associations between religious affiliations and specialties in general. To address this anecdotal hypothesis, we investigated the diversity of religious affiliation in specialist medical workforces, with a focus on specialties in secondary care. We drew on freedom of information data from the General Medical Council (GMC) that shows a snapshot of specialists on the register, and Health Education England (HEE) on trainee applications which would influence the future makeup of the specialists. It may be argued that evaluating the influence of religion in healthcare decisions should be prioritised, especially considering the aforementioned findings of the clinician-patient concordance literature. This study contributes to the debate on physician workforce diversity by focusing on the protected characteristic of religion across medical specialties in the UK.

## 2. Methods

### 2.1. Objectives

To investigate the specialist medical workforce makeup (doctors who have completed training) by religious affiliation, and to investigate trends in applications to training programmes by trainee doctors by religious affiliation.

### 2.2. Hypothesis

Anecdotal reports by trainees suggested that there were fewer than expected Muslims doctors in Anaesthesia and Intensive Care in the UK. We therefore investigated the possibility of hitherto unknown associations between all available religious affiliations and specialties. We hypothesised that if we found any robust associations, that they should not be confounded by ethnicity or foreign medical school training.

### 2.3. Data

The Freedom of Information (FOI) Act 2000 provides the public with the right to access information held by public authorities within the UK, including the National Health Service (NHS).

We looked at the faith of specialists in the UK in 2019 through FOI requests to the GMC. We also requested FOI data from HEE on trainee applications to specialties for a single year in August 2019. For the HEE data, we included only training programmes that attract a national training number (NTN), such that their completion leads to registration on the specialist register. We merged the specialties with NTNs in the HEE data to match those from the GMC categories. The list of the investigated specialties and faiths are shown in Tables 1 and 2.

**Table 1:** GMC data on specialist registrations by religious affiliation (2019). The percentage in the brackets demonstrates the percentage of each religious affiliation within that specialty as a total of the religious affiliation i.e., 9.7% of all atheists are anaesthetists or intensivists. Please note that each doctor can be registered in more than one speciality.

	Religious Affiliations, n (%)										
Specialties	Atheism	Buddhism	Christianity	Hinduism	Islam	Judaism	Sikh	Other	prefer not to say	Unknown	Total
Anaesthesia and Intensive Care	1278 (9.7%)	45 (7.7%)	1537 (7.6%)	486 (9.7%)	197 (4.3%)	31 (5.7%)	25 (4.8%)	38 (6.7%)	379 (8.0%)	6471 (7%)	10487
Emergency Medicine	369 (2.8%)	10 (1.7%)	449 (2.2%)	96 (1.9%)	102 (2.2%)	11 (2.0%)	7 (1.4%)	6 (1.1%)	79 (1.7%)	1279 (1.4%)	2408
General Practice	5225 (39.8%)	174 (29.6%)	7632 (37.6%)	1364 (27.1%)	1789 (38.8%)	175 (32.2%)	282 (54.2%)	187 (32.9%)	1936 (41.0%)	44713 (48.5%)	63477
Medicine	2116 (16.1%)	141 (24%)	3350 (16.5%)	845 (16.8%)	856 (18.6%)	121 (22.3%)	58 (11.2%)	93 (16.3%)	709 (15.0%)	13036 (14.1%)	21325
Obstetrics & Gynaecology	334 (2.6%)	19 (3.2%)	806 (4.0%)	292 (5.8%)	190 (4.1%)	12 (2.2%)	11 (2.1%)	16 (2.8%)	121 (2.6%)	2269 (2.5%)	4070
Occupational medicine	68 (0.5%)	0 (0%)	120 (0.6%)	9 (0.2%)	8 (0.2%)	2 (0.4%)	0 (0%)	1 (0.2%)	30 (0.6%)	329 (0.4%)	567
Ophthalmology	181 (1.4%)	14 (2.4%)	404 (2.0%)	105 (2.1%)	100 (2.2%)	9 (1.7%)	8 (1.5%)	12 (2.1%)	102 (2.2%)	1406 (1.5%)	2341
Paediatrics	585 (4.5%)	38 (6.5%)	1120 (5.5%)	446 (8.9%)	204 (4.4%)	35 (6.5%)	9 (1.7%)	34 (6.0%)	185 (3.9%)	3336 (3.6%)	5992
Pathology	314 (2.4%)	21 (3.6%)	403 (2.0%)	120 (2.4%)	87 (1.9%)	16 (3.0%)	7 (1.4%)	10 (1.8%)	123 (2.6%)	1912 (2.1%)	3013
Psychiatry	936 (7.1%)	51 (8.7%)	1074 (5.3%)	426 (8.5%)	301 (6.5%)	56 (10.3%)	37 (7.1%)	63 (11.1%)	345 (7.3%)	4919 (5.3%)	8208
Public health	139 (1.1%)	1 (0.2%)	168 (0.8%)	16 (0.3%)	20 (0.4%)	7(1.3%)	2 (0.4%)	8 (1.4%)	34 (0.7%)	652 (0.7%)	1047
Radiology	519 (4.0%)	36 (6.1%)	822 (4.1%)	251 (5.0%)	215 (4.7%)	22 (4.1%)	19 (3.7%)	30 (5.2%)	244 (5.2%)	3853 (4.2%)	6011
Surgery	1142 (8.7%)	41 (7.0%)	2531 (12.5%)	597 (11.9%)	565 (12.3%)	49 (9.0%)	55 (10.6%)	76 (13.4%)	470 (10.0%)	8533 (9.3%)	14059
Other	10 (0.1%)	1 (0.2%)	16 (0.1%)	2 (0.0%)	3 (0.1%)	1 (0.2%)	1 (0.2%)	0 (0%)	7 (0.2%)	35 (0.0%)	76
Total individuals	13117 (100%)	587 (100%)	20316 (100%)	5037 (100%)	4609 (100%)	543 (100%)	520 (100%)	569 (100%)	4724 (100%)	92271 (100%)	142293

**Table 2:** HEE data on trainee doctor applications to specialties with national training numbers by religious affiliation (2019). The specialties in this HEE data table were devised to reflect that of the GMC classification, using all training posts with national training numbers. For example as the GMC used Anaesthetics and intensive care as one group, these were combined for the HEE data.

	Religious Affiliations									
Specialties	Atheism (n)	Buddhism (n)	Christianity (n)	Hinduism (n)	Islam (n)	Judaism (n)	Sikh (n)	other (n)	not disclosed (n)	Total (n)
Anaesthesia and Intensive Care	307 (10.0%)	12 (2.5%)	239 (4.8%)	71 (4.9%)	70 (1.7%)	6 (6.7%)	9 (5.3%)	40 (5.4%)	153 (5.4%)	907
Emergency medicine	300 (9.8%)	24 (4.9%)	283 (5.6%)	103 (7.1%)	259 (6.1%)	6 (6.7%)	10 (5.9%)	48 (6.5%)	166 (5.9%)	1199
General Practice	902 (29.3%)	149 (30.7%)	2060 (41%)	533 (36.8%)	1579 (37.3%)	17 (19.1%)	65 (38.2%)	247 (33.6%)	975 (34.4%)	6527
Medicine	493 (16.0%)	184 (37.9%)	675 (13.4%)	235 (16.2%)	949 (22.4%)	20 (22.5%)	30 (17.6%)	100 (13.6%)	458 (16.2%)	3144
Obstetrics & Gynaecology	131 (4.3%)	27 (5.6%)	324 (6.4%)	101 (7.0%)	275 (6.5%)	6 (6.7%)	9 (5.3%)	32 (4.3%)	117 (4.1%)	1022
Occupational medicine	2 (0.1%)	0 (0%)	12 (0.2%)	2 (0.1%)	3 (0.1%)	1 (1.1%)	0 (0%)	3 (0.4%)	2 (0.1%)	25
Ophthalmology	49 (1.6%)	9 (1.9%)	91 (1.8%)	19 (1.3%)	77 (1.8%)	3 (3.4%)	3 (1.8%)	21 (2.9%)	84 (3.0%)	356
Paediatrics	152 (4.7%)	16 (3.1%)	306 (5.6%)	73 (3.9%)	250 (4.3%)	6 (6.7%)	4 (2.4%)	36 (4.8%)	114 (3.6%)	957
Pathology	33 (1.1%)	2 (0.4%)	52 (1.0%)	13 (0.9%)	31 (0.7%)	2 (2.2%)	0 (0%)	13 (1.8%)	38 (1.3%)	184
Psychiatry	133 (4.3%)	17 (3.5%)	166 (3.3%)	39 (2.7%)	101 (2.4%)	0 (0%)	6 (3.5%)	33 (4.5%)	88 (3.1%)	583
Public health	196 (6.4%)	8 (1.6%)	260 (5.2%)	36 (2.5%)	59 (1.4%)	2 (2.2%)	6 (3.5%)	67 (9.1%)	139 (4.9%)	773
Radiology	150 (4.9%)	23 (4.7%)	185 (3.7%)	93 (6.4%)	253 (6.0%)	3 (3.4%)	13 (7.6%)	39 (5.3%)	208 (7.3%)	967
Surgery	232 (7.5%)	15 (3.1%)	397 (7.9%)	146 (10.1%)	391 (9.2%)	17 (19.1%)	15 (8.8%)	57 (7.7%)	301 (10.6%)	1571
Other	1 (0.0%)	0 (0%)	1 (0.0%)	2 (0.1%)	1 (0.0%)	0 (0%)	0 (0%)	1 (0.1%)	1 (0.1%)	7
Total individuals	3081 (100%)	486 (100%)	5051 (100%)	1466 (100%)	4298 (100%)	89 (100%)	170 (100%)	737 (100%)	2844 (100%)	18222 (100%)

We also obtained data from the GMC on potential confounders, including ethnicity and whether doctors were trained in medical schools in the UK or elsewhere.

The GMC data is collected by the GMC at the time of registration when one graduates and first registers with the GMC. It can be updated at any time, including at time of renewal. It is not mandatory, thus there is a large unknown cohort. The HEE data is collected at time of application and is mandatory, but applicants can opt to not disclose their religious affiliation.

### 2.4. Statistical analysis

Due to a low prior probability of finding a genuine significant association and to avoid false positives, we chose a reduced statistical significance threshold of 0.005 [44] and performed conservative global Bonferroni corrections for multiple comparisons using Chi-squared tests, as well as normalised mutual-information scores.

### 2.5. Sensitivity analyses

As faith is self-declared, we report effect-sizes on data for which the doctors’ religious affiliations were known. Nevertheless, we investigated the robustness of all findings through multiple sensitivity analyses to the missing data, including any changes to the strength of associations. Sensitivity analyses involved imputing missing values based on UK doctors’ data [45] or the England and Wales general population data [46] and including or excluding missing data (see S1 Appendix for full details on sensitivity analyses).

### 2.6. Reporting and subgroup analyses on potential confounders

We report on the associations that were robust on all seven sensitivity analyses, with 99.5% confidence intervals. Where a robust association was found, we investigated whether ethnicity or having a foreign primary medical qualification could explain the underlying association.

## 3. Results

### 3.1. GMC and HEE data

GMC provided data on 142293 doctors in thirteen different specialties: Anaesthesia and Intensive Care, Emergency Medicine, General Practice, General Medicine, Obstetrics and Gynaecology, Occupational Medicine, Ophthalmology, Paediatrics, Pathology, Psychiatry, Public health, Radiology, Surgery, and other; and ten different religious affiliations: Atheism, Buddhism, Christianity, Hinduism, Islam, Judaism, Sikhism, “other”, “prefer not to say”, and “unknown” (Table 1). HEE provided data on 18222 applications (Table 2). The specialties and religious affiliations in the HEE applications were matched to reflect the GMC data.

### 3.2. Associations between doctors’ faiths and specialties

#### 3.2.1 A look at all faiths and specialties

Chi-squared tests found significant association between religious affiliation and doctors’ specialty (GMC, p < 0.0001). There was also a significant association between religious affiliation and doctors applying to specialties in the HEE data (p < 0.0001). We therefore explored the pair-wise association between doctors of specific faiths and all specialties for both GMC and HEE data.

**Fig 1:**
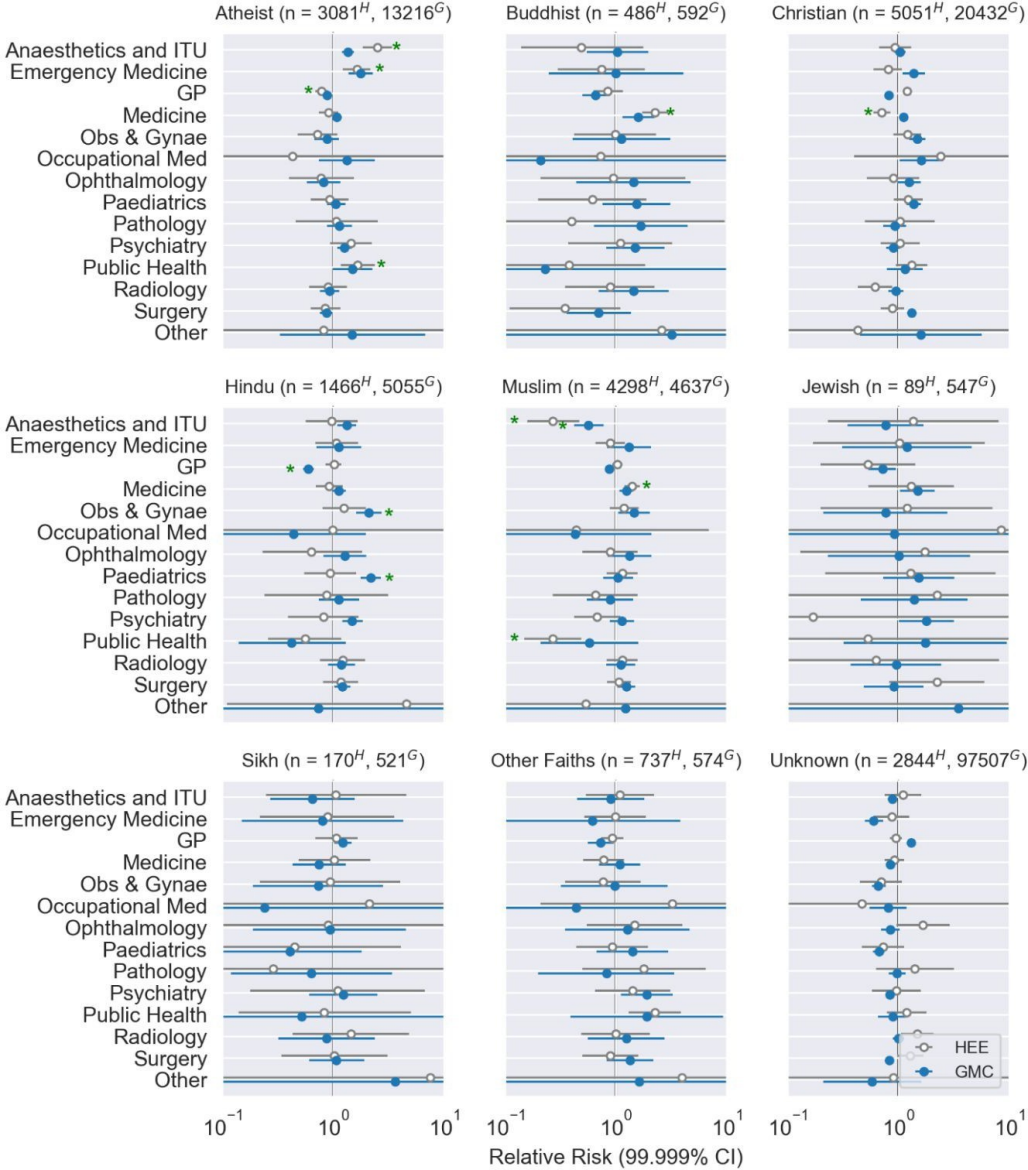
Associations between religious affiliations and specialty. As alpha significance threshold was 0.5%, after Bonferroni correction we show 99.999% confidence intervals. Green stars signify that the association persisted across all seven sensitivity analyses (details in the S1 Appendix). Fewer Muslim anaesthetist and Intensive Care applicants in 2019 and consultants in 2020 were the only associations that were robust in both GMC and HEE datasets.

Fig 1 shows the point estimates and confidence intervals of the likelihoods of junior doctors applying to various specialties (HEE data in grey) and the likelihood of being consultants in specific specialties (GMC data in blue), broken down by faith.

Statistically significant associations in the HEE applications that were robust to all seven sensitivity analyses are shown in Fig 1, and included the following:

- Compared to doctors of all other faiths, Atheist junior doctors were:
   ⚬ two-and-a-half times more likely to apply for Anaesthesia and Intensive Care training (RR 2.51 99.5% CI [2.08, 3.04])
   ⚬ more likely to apply to Public Health (RR 1.67 [1.33, 2.09])
   ⚬ more likely to apply to Emergency Medicine (RR 1.64 [1.37, 1.96])
   ⚬ less likely to apply to General Practice (RR 0.79 [0.72, 0.86])
- Compared to doctors of all other faiths, Buddhist junior doctors were:
   ⚬ more likely to apply to Medicine (RR 2.27 [1.91, 2.69])
- Compared to doctors of all other faiths, Christian doctors were:
   ⚬ less likely to apply for Medicine (RR 0.71 [0.64, 0.8])
- Compared to doctors of all other faiths, Muslim junior doctors were:
   ⚬ less likely to apply to Anaesthesia and Intensive Care (RR 0.27 [0.19, 0.38])
   ⚬ Less likely to apply to Public Health (RR 0.27 [0.18, 0.39])
   ⚬ and more likely to apply to Medicine (RR 1.4 [1.27, 1.54])

The strengths of these associations for the HEE data were robust whether we included or excluded missing data.

Significant results in GMC specialist registrations that were also robust to all seven sensitivity analyses included the following (Fig 1):

- Compared to doctors of all other faiths, Hindus were:
   ⚬ less likely to be GPs on the specialist register (RR 0.6 [0.56, 0.64])
   ⚬ more likely to be consultant Obstetricians (RR 2.11 [1.79, 2.49])
   ⚬ more likely to be consultant Paediatricians (RR 2.2 [1.92, 2.51])
- Compared to doctors of all other faiths, Muslims were:
   ⚬ less likely to be Anaesthetic and Intensive Care consultants (RR 0.57 [0.47, 0.7])

Again, the strength of these associations for the GMC data were relatively robust to sensitivity analyses on missing data.

**Fig 2:**
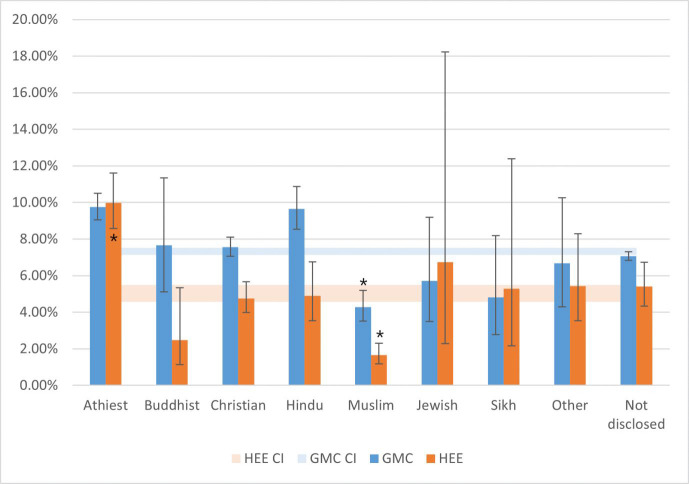
Anaesthetics and intensive care by religious affiliation. Percentage of doctors of each faith that were anaesthetists or intensivists on the specialist register in 2019 (blue bars) or applied to the specialties in 2019 (orange bars). Horizontal bands indicate the percentage of all consultants in anaesthesia or intensive care (blue), and percentage of all trainee doctor applications to these specialties in 2019 (orange) with Wilson 99.5% confidence intervals. In the case of proportional representation, one would expect overlap of confidence intervals of bars with the band of the same colour, which does occur in most cases, except for Atheists (GMC and HEE), Hindus (GMC only), and Muslims (GMC and HEE). While Atheists and Hindus are overrepresented, Muslims are underrepresented. * = robust association present on all sensitivity analyses (see S1 Appendix for full details). For example, 9.7% of all atheists on the specialist register are within Anaesthesia or Intensive care (blue bar), while 7.3% (±99.5% CI) of all doctors are anaesthetists or intensivists (horizontal blue band). With proportional representation, one would expect religious groups to have similar percentage representation within Anaesthesia and Intensive care.

Therefore, the only significant and robust association that was present on both the GMC and HEE datasets (affecting the same religious group and specialty), was disproportionately fewer Muslim Anaesthesia & Intensive Care doctors than expected, both as consultants (RR 0.57 [0.47, 0.7]) and trainee applicants (RR 0.27 [0.19, 0.38], Figs 1 and 2). Additionally, the largest strength of association for both GMC and HEE data was between Muslim Anaesthetists and Intensivists.

#### 3.2.2 Focus on Anaesthesia and Intensive Care

Fig 2 summarises the proportions of doctors of different faiths in Anaesthesia and Intensive Care. 7.3% of 142293 UK specialists were Anaesthetists and/or Intensivists, while only 4.3% of all Muslims were Anaesthetists and/or Intensivists. 5.0% of all 18222 applications in 2019 (HEE) were made to Anaesthesia and Intensive Care, only 1.63% of Muslims made applications to this specialty (Fig 2). Compared to all other faiths, Muslim doctors were 73% less likely to apply to Anaesthesia and Intensive Care than any other specialty (RR 0.27 99.5% CI [0.19, 0.38], p = 1.2x10^-30^), and there were nearly half (58%) as many Muslim consultant anaesthetists and/or Intensivists on the GMC register as expected (RR 0.57 99.5% CI [0.47, 0.7], p = 3.5x10^-16^).

There were other associations amongst specialties and faiths which are highlighted in the S1 Appendix, including a greater than expected number of Atheist and Hindu Anaesthesia and Intensive Care specialists on the GMC register, but these were not robust to all sensitivity analyses (Fig 2).

### 3.3. Missing data

There were missing (“unknown” or not declared) religious affiliations in 15% and 68% of the HEE and GMC data, respectively. Ophthalmology (24%) and GP (73%) had the most missing values in the HEE and GMC data, respectively. However, missing data on religious affiliation was not significantly associated with doctors’ specialty (p>0.99), similar to previous studies analysing voluntarily reported protected characteristics [47].

### 3.4. Subgroup analyses for confounders

Subgroup analysis was conducted on the GMC dataset for the inverse association between being a Muslim doctor and being a consultant in Anaesthesia or Intensive Care. We looked at subgroup information on ethnicity and whether doctors had a primary UK or foreign medical qualification. This was performed posthoc on an updated 2021 GMC dataset, the data values and proportion of doctors in each specialty were much like the 2019 GMC data.

Both foreign and UK trained Muslim doctors were equally less likely to be Anaesthesia and Intensive Care consultants, and there was no significant difference between these two subgroups (RR 0.54 99.5% CI [0.44, 0.66]; RR 0.56 99.5% CI [0.49, 0.63] respectively). Amongst the ethnic groups, the only significant association between Muslim doctors and Anaesthesia and Intensive Care was in Asians (RR 0.48 99.5% CI [0.42, 0.55]). When considered univariately, Asian doctors, irrespective of their faith, were less likely to be Anaesthesia and/or Intensive Care consultants (RR 0.84 99.5% CI [0.81, 0.88]). Thus, overall, Asians were 16% less likely to be Anaesthetists or Intensivists whereas Muslims or Muslim-and-Asian doctors were 43% and 52% respectively less likely to be consultants in these specialties, suggesting that being Muslim might be an important independent factor in explaining the association.

## 4. Discussion

The analysis demonstrates that there are robust, and unexplored faith-specialty associations, the strongest of which is disproportionately fewer Anaesthetic & Intensive Care consultants on the GMC specialist register with a”Muslim” religious affiliation. This association was not explained by being foreign or UK-trained. Additionally, although the association was present for Asian-Muslims when looking at ethnic subgroups, the strength of the association was significantly larger for Muslim doctors than Asian doctors. Overall, Asians were 16% less likely to be Anaesthetists and Intensivists whereas Muslim doctors were 43% and Muslim-Asian doctors 52% less likely to be anaesthetic or intensivist consultants. The significance of having fewer than expected applicants to an already lacking Muslim workforce within Anaesthesia and Intensive Care means that this trend is likely to continue into the future until it is addressed. Although there were other associations between religions and specialties, for example, the greater than expected numbers of Hindu and Atheist anaesthesia and intensive care consultants, these were not robust to all sensitivity analyses.

These results suggest that there might be hitherto unknown underlying barriers to entry that prevent doctors with a Muslim religious affiliation to choose and practice in this specialty. Religion may be important in determining specialty choice due to moral conflict, socio-cultural and historical barriers to entry, or lack of role models; however, empirical research is required to illuminate this area of ambiguity.

The large proportions of missing data with both data sets should not be ignored. The GMC data was missing 68%, while the HEE data was missing 15% in accurate religious affiliation. However, the missing data were not significantly associated with specialty. The authors do not believe this could explain the robust association between Muslims and anaesthesia and intensive care, as they were consistent despite multiple sensitivity analyses and because the analysis was hypothesis driven from the start. Nevertheless, the associations do not determine causality, nor are they evidence of discrimination.

Provision of equity-oriented health care leads to improved patient outcomes over time [48]. Diversity of representation amongst specialty doctors is a requirement for health equity [47], as specialties with diverse senior clinicians are more likely to be able to address the needs of disadvantaged communities [49]. Although many studies have focused on diversity of sex and ethnic backgrounds, this is the first study to consider the protected characteristic of religion as a potential explaining factor of disparities related to workforce diversity. Ethnic and cultural barriers between physician and patient, especially in specialties that deal with more pronounced ethically contentious dilemmas, can result in trust and compliance barriers and cultural disparities in health, potentially affecting health outcomes [23, 50]. Napier, Ancarno [13] provide a comprehensive critique of the uptake of cultural competence in practice. They highlight the dangers of superficial and stereotypical solutions in health systems, and the many complex facets which are not often adequately addressed in order to be effective in nurturing communication between physician and patient in order to remove barriers to care.

Anesthetists and Intensivists work at the boundaries of various medical and ethical challenges. They are regularly involved with informed consent for surgery and anaesthesia, blood transfusions, organ donation and retrieval, critical illness, end-of-life decisions, and decisions of ceilings of care [51]. For many of these issues, medicine is not the only dominant voice. Lay knowledge and culture, alongside religious beliefs and practices, often compete with medical knowledge to influence and direct decisions [52–55]. Other areas of the Anesthetist’s regular work that can interface with religious influence include dealing with preoperative anxiety which can affect surgical outcomes [56, 57]. Various studies from the US have documented minorities being more likely to die in intensive care, that were not explained by socioeconomic status [58–60]. Moreover, there is evidence of more nuanced minority inequalities in intensive care practices, including lower non-verbal clinician-patient and clinician-surrogate communication scores [18, 61], higher conflict with clinicians over treatment choices [62, 63] and lower tracheostomy prioritization [64]. Considering the extensive literature on the benefits of clinician-patient cultural concordance, it can be argued that in multicultural societies, physician workforce diversity and adequate representation of various cultures and religious beliefs across medical specialties are imperative for improving health outcomes. In specialties with more pronounced ethically contentious dilemmas such as Anaesthesia & Intensive Care, diversity may help alleviate some of the challenges of the conflicting voices that feed into difficult decisions by patients and their families, and can potentially lead to more comprehensive ‘informed consent’ and treatment concordance. The focus on this specialty is topical, as Anesthetist and Intensivists have been key stakeholders in the critical care of COVID-19 patients [65, 66], being at the centre of dealing with the complexity of critical care decisions under circumstances of a global pandemic, and widespread cultural/religiously influenced skepticism to vaccination and treatment of COVID-19.

Following Schram, Boyd-Caine [67] who argue for incorporating law into the understanding of the social determinants of health and addressing of health equity, this study argues for a comprehensive programme of research to study the influence of religion and religious representation in medical specialties on health equity. The findings in this study, along with concerns for adequate cultural competence and clinician-patient relationships to improve health outcomes, support the argument that religious diversity in Anaesthesia and Intensive Case specialty needs to be further evaluated. This study is in line with findings from the USA that demonstrated an under-representation of minorities in Anaesthesia & Intensive Care [51], further suggesting that more comprehensive research is needed at both levels of specialty choices by training physicians, and potential structural and socio-cultural barriers to entry. There is evidence that minority physicians who are under-represented in medical specialties may be more likely to practice in under-served areas and care for minority populations [68, 69]. Thus, strategies to improve physician workforce diversity and specialty training of under-represented groups such as Muslims in Anaesthesia and Intensive Care may entail the added benefit of geographical targeting to reduce health inequalities, as well as addressing calls for contextually-tailored care [48]. Furthermore, this study suggests crucial areas for future research, including exploring the antecedents to specialty selection and whether religion is in fact a direct variable in this decision.

The Royal College of Anaesthetics (RCoA) has recognised that ethnic minorities are at risk of discrimination and have made numerous pledges to defend all protected characteristics specifically collecting data on ethnic minorities within the specialty. Although there is a specific mention of all protected characteristics, only data on ethnicity is being monitored [70]. The RCoA is also monitoring and publishing black and minority ethnic representation within positions of responsibility [71], but again have not explored other protected characteristics. Furthermore, there has been other individuals who have recognised that this specialty has under represented from ethnic minorities [72–73], thus this is an area that needs to be further investigated and addressed by both the RCoA and Faculty of Intensive Care Medicine.

### 4.1. Limitations

The limitations of our study include significant unknowns, especially in the GMC dataset (Table 1), where 68% of faiths were unknown. We tried to mitigate this using multiple imputation methods and sensitivity analyses. For comparison, 39% of doctors’ faiths were either unknown or documented as “other” in the larger UK doctors’ workforce, with a similar proportion of missing data in the general population, but ethnicity data in the 2011 census is only in missing in 8%.

Additionally, we only looked at HEE data for a single application year in 2019 which showed a dramatic overrepresentation of Muslim junior doctor applicants to all specialties (23.6%) compared to the 3.7% of UK doctors that are Muslim3. Although we checked for confounders by foreign training and ethnicity, we did not check for age and sex as potential confounders. We emphasise that we have shown associations, and this should not be taken to imply causation.

Furthermore, there are other routes that individuals can take to achieve registration as a specialist on the GMC register. The Certificate of Eligibility for Specialist Registration (CESR) is one. This route is often taken by those that have international qualifications, therefore this route may constitute a disproportionate number of applicants from minority ethnic and religious groups.

### 4.2. Conclusions

We found that medical doctors with a ‘Muslim’ religious affiliation were significantly underrepresented in Anaesthesia and Intensive care at both the senior clinician level and in trainee applications. Religion is a ‘protected characteristic’ as defined by the UK Equalities Act 2010 and the US Civil Rights Act 1964, which is a fundamental characteristic of ethnicity and a significant component of many people’s identity. Religion can influence people’s culture, health beliefs, behaviours, treatment decisions, and consequently health outcomes. Discussion and decisions around ethically contentious issues in patient treatment and care can be facilitated by minority representation in specialties such as Anaesthesia and Intensive Care. This is also in line with calls for a comprehensive evaluation of the effectiveness of cultural competence training, and more targeted ‘cultural-religious competence’ to be built into the training and development of physicians.

It is important to further explore potential historical and socio-cultural barriers to entry of training medics into under-represented specialties such as Anaesthesia and Intensive Care. Improving physician workforce diversity more holistically can have the added benefit of reducing health inequalities by improving access to services and reach to under-served areas. Having proportional representation amongst specialties provides a platform for minorities to represent patient groups and shape debates related to the specialty, such as that of end-of-life care. This is particularly pertinent at the senior clinician level. Further evaluations are needed to assess the persistence of these religious associations and to explore their antecedents.

## Supporting information

S1 Appendix (DOCX)

S1 Data (XLSX)

S2 Data (XLSX)
