# Supplementary material for: Workforce diversity in specialist physicians: Implications of findings for religious affiliation in Anaesthesia & Intensive Care
Source: PLoS One. 2023 Aug 23;18(8):e0288516. doi: 10.1371/journal.pone.0288516 (PMC10446200; doi:10.1371/journal.pone.0288516)
Supplement: S1 Appendix — (DOCX) [file pone.0288516.s001.docx]

# Supplementary Materials

# Faith-Based Representations in UK Specialties

Ali Alim-Marvasti* ^1^, Mohammed Jawad ^2^, Chibueze Ogbonnaya ^3^, Ali Naghieh ^4,5^

1 UCL Queen Square Institute of Neurology, University College London, UK

2 Imperial College Healthcare NHS Trust, London, UK

3 Institute of Child Health, University College London, UK

4 School of Public Policy, University College London, UK

5 Middlesex University Business School, UK

*Corresponding author: [alijesus.alim-marvasti@nhs.net](mailto:alijesus.alim-marvasti@nhs.net), [a.alim-marvasti@ucl.ac.uk](mailto:a.alim-marvasti@ucl.ac.uk)

## Table of Contents

[1. Supplementary Methods 3](#_Toc70092770)

[1.1. Data Tables 3](#_Toc70092771)

[1.2. Multiple tests of proportions 6](#_Toc70092772)

[1.3. Bonferroni Correction and significance level 6](#_Toc70092773)

[1.4. Theil’s U (Asymmetric Normalised Mutual Information Score, NMI) 6](#_Toc70092774)

[1.5. Sensitivity Analyses and Keeping Only Robust Results 7](#_Toc70092775)

[2. Supplementary Results and Tables 10](#_Toc70092776)

[2.1. Main Results: Multiple Tests of Proportions with Sensitivity Analysis 11](#_Toc70092777)

[2.2. Normalised Mutual Information (Theil’s U) 13](#_Toc70092778)

[2.3. Results of Further Sensitivity Analyses 16](#_Toc70092779)

[2.4. HEE Robust Result Overlays 19](#_Toc70092780)

[2.5. GMC Robust Result Overlays 22](#_Toc70092781)

[2.6. How many training cohorts would it take to mitigate the deficit in Muslim consultant anaesthetists? 24](#_Toc70092782)

[3. Summary and Conclusions 25](#_Toc70092783)

[4. Data and Code Availability 27](#_Toc70092784)

[5. Supplementary References 28](#_Toc70092785)

## Abbreviations

A&IC: Anaesthesia and Intensive Care

CCT: Certificate of Completion of Training

FOI: Freedom of Information

GMC: General Medical Council

GP: General Practice

HEE: Health Education England

ST: Specialist Training

## Supplementary Methods

Wherever we use Anaesthesia, we mean both Anaesthesia and Intensive Care.

### Data Tables

The data for HEE applications in 2019 to medical specialties, and GMC specialist registrations in 2020 by religious affiliations were both obtained through a FOI request. For HEE data, we only looked at specialties with national training numbers as on completion these would result in CCT certification and entry on the GMC specialist register. We then merged these specialties in the HEE data to match those from the GMC categories. For example, surgery included Cardiothoracic Specialist Trainee year 1 (ST1) run through, Cardiothoracic ST3, General and Vascular, Neurosurgery ST1 run through, Neurosurgery ST3, Oral/Maxillofacial ST1 run through, Oral/Maxillofacial ST3, Otolaryngology, Paediatric surgery, Plastics, Trauma and Orthopaedics and Urology.

Detailed categorisations of the HEE data are included in the repository spreadsheet named “Index_HEE_Categorisation” where the G*rouped data HEE* tab categorises specialties to reflect those in the GMC and produces Table 1 above. To protect doctors’ identities, some cells have “<5” in the original data. These were approximately imputed to between 1 and 4.

The final list of specialties in both GMC and HEE datasets were: Anaesthesia, Emergency Medicine, General Practice (GP), Internal Medicine, Obstetrics and Gynaecology (O&G), Occupational Medicine, Ophthalmology, Paediatrics, Pathology, Psychiatry, Public Health, Radiology, Surgery and other.

The list of religious affiliations was: Atheist, Buddhist, Christian, Hindu, Muslim, Jewish, Sikh, other and unknown; the latter included “prefer not to say”.

The data for HEE applications is shown in Table 1 and for the GMC specialist registrations is shown in Table 2.

The data, and python code to reproduce the analyses are fully available on the repository:

<https://github.com/thenineteen/GMC_HEE_2019>

|  | Atheism | Buddhism | Christianity | Hinduism | Islam | Judaism | Sikh | other | not disclosed | Total |
| --- | --- | --- | --- | --- | --- | --- | --- | --- | --- | --- |
| Anaesthetics and Intensive Care | 307 | 12 | 239 | 71 | 70 | 6 | 9 | 40 | 153 | 907 |
| Emergency medicine | 300 | 24 | 283 | 103 | 259 | 6 | 10 | 48 | 166 | 1199 |
| GP | 902 | 149 | 2060 | 533 | 1579 | 17 | 65 | 247 | 975 | 6527 |
| Medicine | 493 | 184 | 675 | 235 | 949 | 20 | 30 | 100 | 458 | 3144 |
| Obs&gyne | 131 | 27 | 324 | 101 | 275 | 6 | 9 | 32 | 117 | 1022 |
| Occupational medicine | 2 | 0 | 12 | 2 | 3 | 1 | 0 | 3 | 2 | 25 |
| Ophthalmology | 49 | 9 | 91 | 19 | 77 | 3 | 3 | 21 | 84 | 356 |
| Paediatrics | 152 | 16 | 306 | 73 | 250 | 6 | 4 | 36 | 114 | 957 |
| Pathology | 33 | 2 | 52 | 13 | 31 | 2 | 0 | 13 | 38 | 184 |
| Psychiatry | 133 | 17 | 166 | 39 | 101 | 0 | 6 | 33 | 88 | 583 |
| Public health | 196 | 8 | 260 | 36 | 59 | 2 | 6 | 67 | 139 | 773 |
| Radiology | 150 | 23 | 185 | 93 | 253 | 3 | 13 | 39 | 208 | 967 |
| Surgery | 232 | 15 | 397 | 146 | 391 | 17 | 15 | 57 | 301 | 1571 |
| other | 1 | 0 | 1 | 2 | 1 | 0 | 0 | 1 | 1 | 7 |
| Total | 3081 | 486 | 5051 | 1466 | 4298 | 89 | 170 | 737 | 2844 | 18222 |

Table 1: HEE data on trainee doctor applications to specialities with national training numbers by religious affiliation (2019). The specialties in this HEE data table were devised to reflect that of the GMC classification, using all training posts with national training numbers. As the GMC used Anaesthetics and intensive care as one group, wherever we refer to anaesthesia, we mean both anaesthesia and intensive care.

|  | Atheist | Buddhism | Christianity | Hinduism | Islam | Judaism | Sikh | other | prefer not to say | unknown | total |
| --- | --- | --- | --- | --- | --- | --- | --- | --- | --- | --- | --- |
| Anaesthesia and ITU | 1278 | 45 | 1537 | 486 | 197 | 31 | 25 | 38 | 379 | 6471 | 10487 |
| Emergency Medicine | 369 | 10 | 449 | 96 | 102 | 11 | 7 | 6 | 79 | 1279 | 2408 |
| GP | 5225 | 174 | 7632 | 1364 | 1789 | 175 | 282 | 187 | 1936 | 44713 | 63477 |
| Medicine | 2116 | 141 | 3350 | 845 | 856 | 121 | 58 | 93 | 709 | 13036 | 21325 |
| Obs&gyne | 334 | 19 | 806 | 292 | 190 | 12 | 11 | 16 | 121 | 2269 | 4070 |
| Occupational medicine | 68 | 0 | 120 | 9 | 8 | 2 | 0 | 1 | 30 | 329 | 567 |
| Opthalmology | 181 | 14 | 404 | 105 | 100 | 9 | 8 | 12 | 102 | 1406 | 2341 |
| Paediatrics | 585 | 38 | 1120 | 446 | 204 | 35 | 9 | 34 | 185 | 3336 | 5992 |
| Pathology | 314 | 21 | 403 | 120 | 87 | 16 | 7 | 10 | 123 | 1912 | 3013 |
| Psychiatry | 936 | 51 | 1074 | 426 | 301 | 56 | 37 | 63 | 345 | 4919 | 8208 |
| Public health | 139 | 1 | 168 | 16 | 20 | 7 | 2 | 8 | 34 | 652 | 1047 |
| Radiology | 519 | 36 | 822 | 251 | 215 | 22 | 19 | 30 | 244 | 3853 | 6011 |
| Surgery | 1142 | 41 | 2531 | 597 | 565 | 49 | 55 | 76 | 470 | 8533 | 14059 |
| other | 10 | 1 | 16 | 2 | 3 | 1 | 1 | 0 | 7 | 35 | 76 |
| total | 13117 | 587 | 20316 | 5037 | 4609 | 543 | 520 | 569 | 4724 | 92271 | 142293 |

Table 2: GMC data on specialist registrations by religious affiliation (2019)

The total columns were provided by the GMC through FOI with the following caveat: a doctor may have more than one specialty and will be counted in each speciality group they appear in, but only once in the total. The difference between the sum of religions (columns) and provided totals is 143081 – 142293 = 0.55% error. This ranged from 0.19% error for Sikh to 0.88% for other religions. We considered this to be acceptable to proceed as if they were mutually exclusive categories.

### Multiple tests of proportions

We used two-by-two chi-squared tests using the chi2_contingency function from python’s scipy.stats module (v 1.31) ^1^, we also had an in built method to use fisher’s exact if the expected numbers were less than 5, but this was not utilised. Only if the results were statistically significant, the normalised ratio was displayed on the heatmap matrix of medical specialties and religious affiliations (as in the Figures below). The normalised ratio for each pair of Specialty/Religious-Affiliation cell values were calculated as below for the heatmaps:

$$Normalised ratio (heatmap)= \frac{pairwise cell value - chi-squared expected value}{chi-squared expected value}$$

The heatmap figures therefore show statistically significant results and their direction and proportional magnitude is indicated by a colour bar which shows their normalised ratio.

### Bonferroni Correction and significance level

Bonferroni correction calculations were performed globally using the following calculation for number of analyses:

- (7x14)x2 [excluding unknowns and other] +
- (9x14)x2 [including other and unknowns] +
- (8x14)x2 [imputed redistribution of unknown data using UK doctors’ data and merging the other & unknown categories]

= 672.

At the 0.5% level of significance, (alpha threshold 0.005), this is equivalent to p<7.4x10^-6^. We used this reduced threshold of 0.5% instead of 5%, as we were after robust results representative of the current and future (for HEE applications) medical workforces in the UK, we wanted to reduce false positive rates and we thought there was a low prior-odds of medical specialities and religious affiliations being significantly correlated. This reduction of the alpha threshold has been suggested in previous studies ^2,3^. Wherever we quote p-values, we use the uncorrected value and thus anything less than 7.4x10^-6^ is considered statistically significant after correction.

### Theil’s U (Asymmetric Normalised Mutual Information Score, NMI)

With the chi-squared analyses, we can’t know whether being of a particular faith determines the specialty or the other way round. But by using an asymmetric normalised mutual information measure (NMI, Theil’s U), we can try to elucidate Bayesian directionality and determine how much information knowing the religion or specialty gives us about the other. Conversely, NMI does not inform us whether the faith and specialty are proportional or inversely proportional, whereas the normalised chi-squared heatmaps do show this.

This is a similar measure to Cramer’s V in terms of use in categorical variables and output between [0, 1]. It is, however, an asymmetric conditional measure, and thus the heat map of association is not symmetric. This is useful when we are interested in bilateral nominal associations. It uses the concept of information entropy.^4^

We take samples from the two discrete variables, faith (X) and specialty (Y). We construct the joint distribution P_X,Y_(x,y) and using Bayesian statistics we calculate the conditional distributions:

P_Y|X_(y|x)= $\frac{P_{Y,X}\left( y ꓵ x \right)}{P_{X}\left( x \right)}$

Similarly repeated for the reverse conditional probability, P_X|Y_(x|y).

We then calculate the entropies of the distributions using Shannon’s information theory:

$$H(X)=-\sum_{x} P_{X}(x). log(P_{X}(x))$$

To find the amount of information of Y that is explained by X (information gain), we subtract the conditional entropy from the prior entropy:

Information gain (Y,X) = H(Y) – H(Y|X)

We can now normalise this. Many measures of NMI use an arithmetic or geometric average of H(X) and H(Y), which renders the NMI a symmetric measure. Others, such as Theil’s U or asymmetric NMI, normalise based on the target variable: in our case, H(specialty).

Asymmetric NMI (Theil’s U) (Y|X) = $\frac{Information gain (Y,X)}{H(Y)}$= $\frac{H(Y) - H(Y|X)}{H(Y)}$

In order to assess Theil’s U for the 2019 HEE data, we first excluded unknowns and other faiths as we were interested in the declared religious affiliations for which data was available (no sensitivity analyses were performed for Theil’s U). We melted the pandas DataFrames with the religious affiliations and specialty applications as column categories, and rows as individual doctors. We then constructed the joint and conditional distributions. The DataFrame was then pivoted to create dummy variables such that each row was a single doctor with multi-one hot encoding of both their religious affiliation and specialty. Asymmetric Theil’s U was then calculated using the python code available on the repository. We did the same for the GMC data, using specialist registrations instead of applications to specialty.

### Sensitivity Analyses and Keeping Only Robust Results

As 15.6% of trainees had not reported their faith when applying to specialties in 2019 (HEE), and 68% of specialists on the GMC specialist register had not done so either in 2020, we performed a total of 7 sensitivity analyses to test for robustness of associations.

It is unclear why there were such a large proportion of unknown religious affiliations especially in the GMC data. These are either due to doctors non-reporting their faith at all, which could introduce self-reporting bias, or possibly due to not collecting the data in the past. Missing data on religious affiliations was not significantly associated with area of doctors’ specialty or HEE applications (p-value>0.99 in each case).

Due to a significant number of unknowns, we performed sensitivity analyses by including (“all data”) and excluding the unknown data (excluding unknowns). We then investigated how imputing the missing religious affiliations by redistributing the unknowns to other categories – based on UK Doctors’ (includes non-specialists) data broken down by faith – would affect results. Specifically, in the absence of further information, we considered the unknowns as a random sample from the UK doctors’ populations (Table 1) and so for each specialty, the unknowns were redistributed based on the religious affiliation proportions.

We preformed the following sensitivity analyses:

1. multiple tests of proportions with all data (as in Tables 1 and 2), including unknown religious affiliations
2. multiple tests of proportions excluding unknown religious affiliations
3. multiple tests of proportions after imputation and redistribution of unknowns based on UK doctors’ data and keeping what remained of the unknown religious category
4. normalised mutual information score (NMI, Theil’s U) excluding other and unknown faiths

After global chi-squared tests, we performed two-by-two chi-squared tests with Bonferroni corrections at the alpha threshold of 0.005.

After performing the above analyses, we overlaid the medical-specialty/religious-affiliation normalised ratio heatmaps and only kept the correlations if the results were robustly present in all four above analyses. Note that Theil’s U is asymmetric and the presence of a predictive value was inspected visually from supplementary Figures 2 and 3.

Post hoc, we further investigated how robust our faith-specialty associations were by:

- altering the method of data imputation (using the England and Wales general population data as a reference instead of the UK doctors’ data), and
- (after data imputation) including or excluding (what was left of) the unknown religious affiliations before performing multiple chi-squared tests, and
- investigating different alpha thresholds of 0.05 and 0.0005 as well as including the post hoc sensitivity analyses in the calculations for Bonferroni correction for multiple comparisons.

In total, we performed four tests investigating sensitivity to imputation methods (using UK doctors’ data or England and Wales general population, each with and without what remained from the unknown category) for each of the HEE and GMC datasets (that is 7 sets of statistical tests for each dataset).

We were only interested in the faith-specialty correlations for declared religions (including atheism), so we excluded “other” and “unknown” religious affiliations in the final overlay of robust associations. For the robust associations, we calculated odds ratios, risk ratios and confidence intervals for both the GMC and HEE data, using two-by-two contingency tables from Tables 1 and 2, with separate calculations for including unknown religious affiliations (Tables 5.1 and 6.1) and excluding unknowns (Tables 5.2 and 6.2). Odds ratios and risk ratios were calculating using a two-by-two matrix, e.g., Muslim junior doctors’ applications to anaesthesia having an odds ratio of 0.25 95% CI [0.20, 0.32] was the ratio of the odds of Muslims applying to anaesthesia (vs other specialties) and the odds of non-Muslims applying to anaesthesia (vs other specialties).

### Data and Code Availability

The GMC and HEE datasets, preprocessing, and Python Jupyter notebook analyses are available on GitHub for full reproducibility:

<https://github.com/thenineteen/GMC_HEE_2019>

## Supplementary Results and Tables

Supplementary Table 3 shows doctors on the specialist register by religious affiliation (GMC data). Supplementary Table 4 shows the same results along with the HEE data in the context of proportions of the general population and all UK doctors.

| **Religious** **Affiliation** | **Proportion of GMC Registered UK Specialists 2019 (%)** |
| --- | --- |
| Atheist | 9.2 |
| Buddhist | 0.41 |
| Christian | 14.28 |
| Hindu | 3.53 |
| Muslim | 3.24 |
| Jewish | 0.38 |
| Sikh | 0.36 |
| Other | 0.40 |
| Unknown and prefer not to say | 68.15 |
| Total | 100 |

Table 3: Proportion of GMC Registered Doctors on the Specialist Register in the UK, 2019.

| **Stats for** | **Atheist** | **Christian** | **Buddhist** | **Hindu** | **Jewish** | **Muslim** | **Sikh** | **Other** | **Unknown** |
| --- | --- | --- | --- | --- | --- | --- | --- | --- | --- |
| England and Wales (2018)^5^ | 42.1% | 46.7% | 0.5% | 1.8% | 0.5% | 5.7% | 0.7% | 1.7% | |
| UK doctors  (2019)^6^ | 19.1% | 34.4% | 0.5% | 2.2% | 0.2% | 3.7% | 1% | 39.1% | |
| **HEE Applications to Specialties (2019)** | 16.9% | 27.7% | 2.7% | 8.0% | 0.5% | 23.6% | 0.9% | 4% | 15.6% |
| **GMC Specialist Register (2019)** | 9.2% | 14.3% | 0.4% | 3.5% | 0.4% | 3.2% | 0.4% | 0.4% | 68.1% |

Table 4: GMC Specialist Register (2019) and HEE Specialty Applications (2019) by religious affiliations, in the broader context of all UK doctors’ population and the general population (England and Wales).

Global chi-squared tests were significant for the HEE data irrespective of sensitivity tests and imputation (Table 1, all data: chi2=1101, p=2.8x10^-166^, dof=104; excluding unknowns and other: chi2=941, p=2.3x10^-148^, dof=78; imputed redistributed using UK Doctors’ proportions all data: chi2=922, p=9.1x10^-138^, dof=91).

Similarly, chi-squared tests were significant for the GMC data (Table 2) (all data: chi2=3010, p=0.0, dof=104; excluding unknowns/other: chi2=1083.9, 6.7x10^-177^, dof= 78; imputed redistributed data using UK Doctors’ proportions: chi2=1109, p<8.2x10^-175^, dof=91).

Two-by-two chi-squared test results for three out of the four analyses are shown in Supplementary Figure 1, and the fourth (Theil’s U) results are show in Supplementary Figure 2 and 3.

### Main Results: Multiple Tests of Proportions with Sensitivity Analysis

Supplementary Figure 1 below shows that the HEE data was relatively robust to including or excluding unknowns and imputation (Figure 1 A, C, D), whereas the GMC data has an atheist column of many significant correlations when the unknowns are excluded (Figure 1B) and similarly many GP row correlations when the unknowns are included (Figure 1D) as the majority of the GP’s faiths were unknown. The majority of both of these are removed by the imputation method using UK doctors’ data to redistribute the unknowns proportionally (Figure 1F). Although this makes it seem like these were artefacts, further sensitivity analysis using different imputation methods show that this may not be the case and suggest that only the GP correlations could be artefacts (Supplementary Figure 4 F, H, J and L).

All Data with UK Doctors’

Imputation / Redistribution

Including all data

Excluding unknown and other faiths

GMC Specialist Registrations Data

HEE Applications to Specialty Data

| A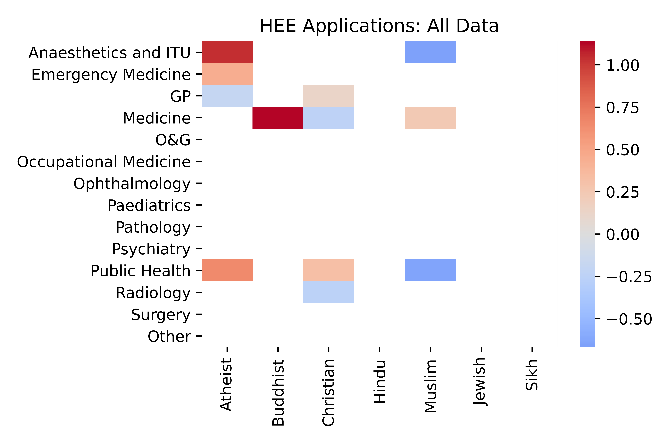 | B  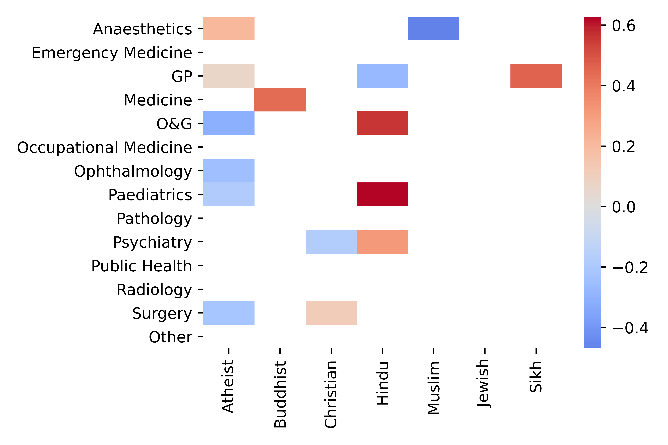 |
| --- | --- |
| C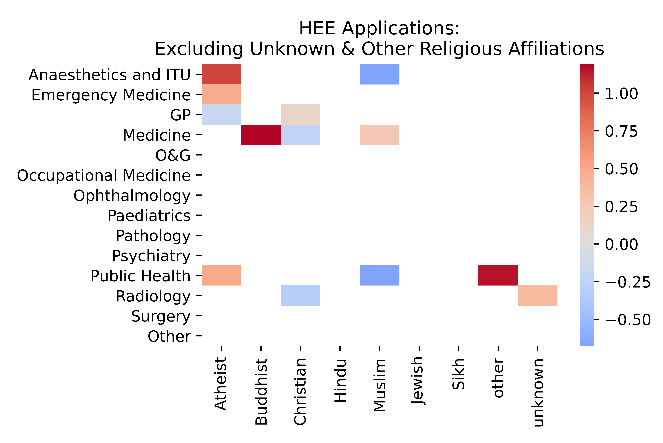 | D  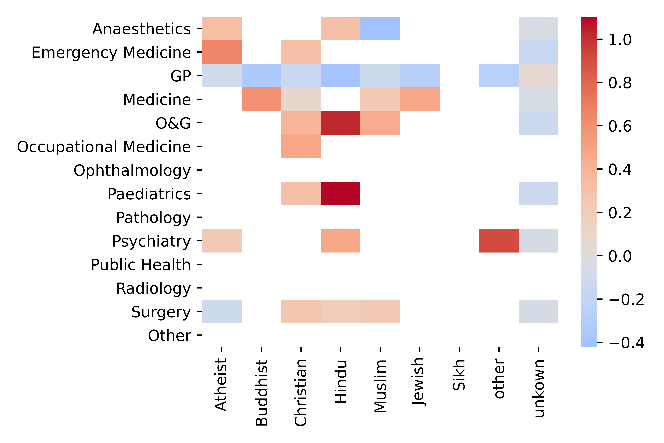 |
| E†  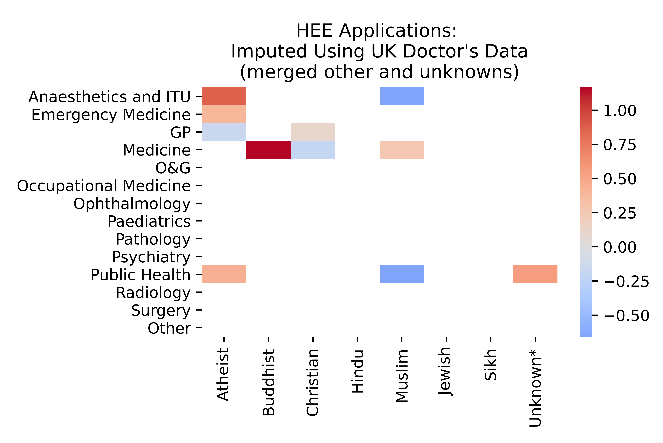 | F†  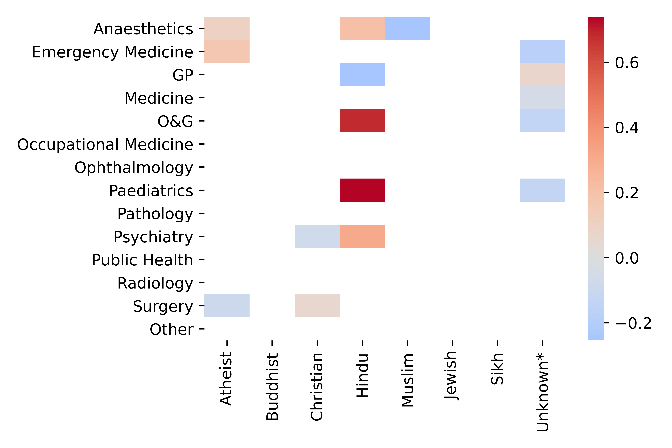 |

***Supplementary Figure 1 Multiple Tests of Proportions:*** *two-by-two chi-squared results for applications to specialty (HEE data, 2019) is represented on the left in green (A, C, E) and for doctors on the specialist register (GMC data, 2019) on the right (B, D, F). A and B represent data analysis excluding unknown religious affiliations. C and D include “other” and unknown religious affiliations. E and F include all data but impute the missing data by redistributing unknowns based on UK doctors’ proportions taken from Table 4. Only the statistically significant (Bonferroni corrected p<0.005) results are shown and the colour bars are normalised to expected frequencies.*

** = As Table 4 contained merged other and unknown religious affiliations, after imputation, these categories are merged.*

*† = further sensitivity analyses based on data imputation methods were performed – see supplementary section on sensitivity analyses* ***section 2.3.2****).*

### Normalised Mutual Information (Theil’s U)

Supplementary Figure 2 shows the results for Theil’s U as a heatmap. As an example of how the results can be interpreted: if we know a doctor is Muslim (“religion_islam” on the x-axis), we have gained more information about whether they will have applied to Anaesthetics & ITU or Public Health than any other specialty. From this figure alone we won’t know if Muslims are *more* or *less* likely to apply to these two specialties, but the chi-squared heatmaps (Supplementary Figure 1) show that Muslims are *less likely* to apply to both Anaesthetics and Public Health. Theil’s U analyses below confirms that around 30% of the variance (information) of Anaesthetics & ITU and Public Health can be determined by knowing the applicant was Muslim, however, the strength of the reverse association is less significant. The results also show two other robust associations: Buddhists applications to Medicine, and Atheist applications to Anaesthetists. These associations are more symmetric than the Muslim ones.


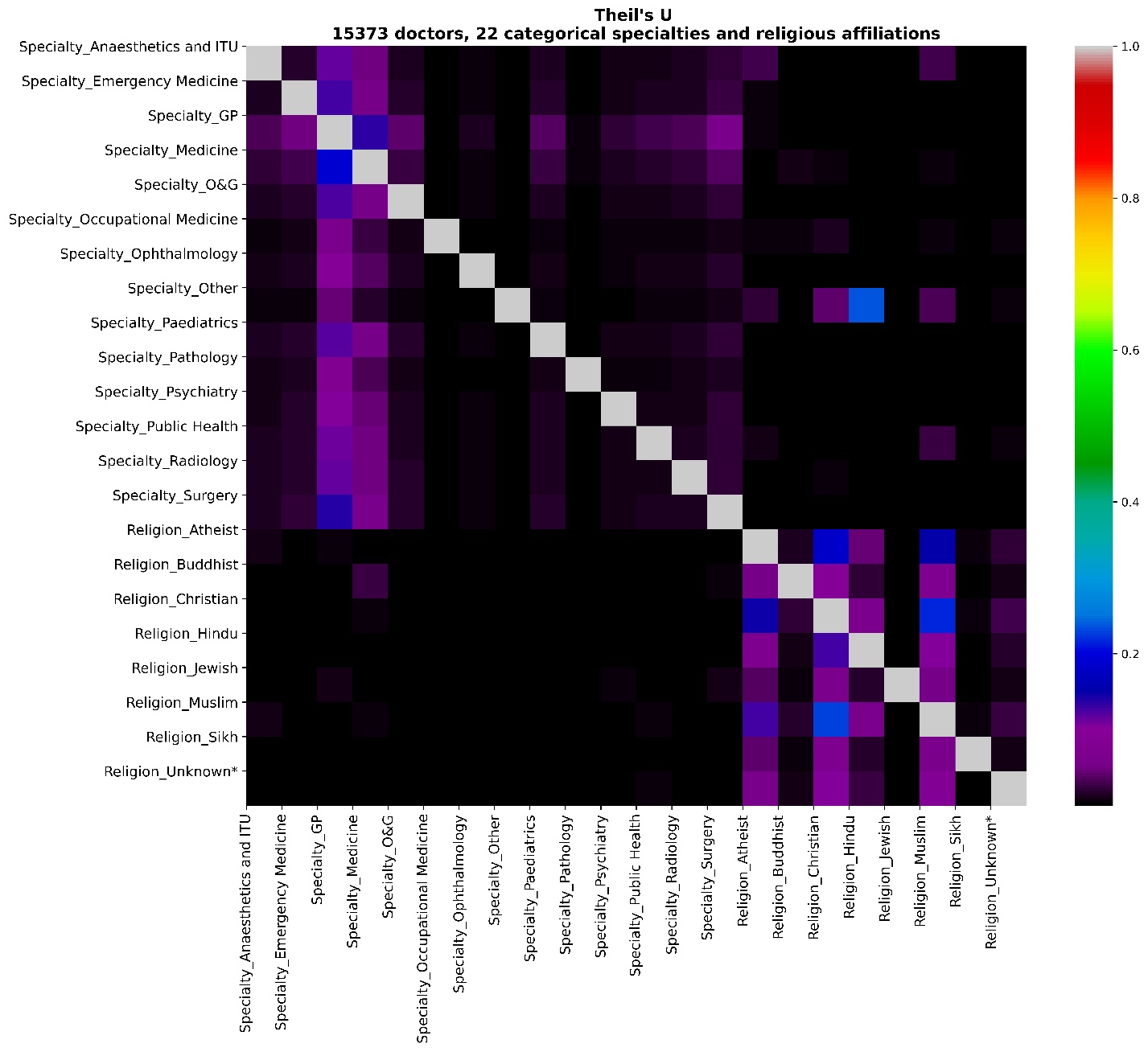


**Supplementary Figure 2:** Theil’s U for all applications to specialties and religious affiliations (HEE 2019, excluding other and unknown faiths). Given knowledge of a variable on the x-axis, the colour bar represents the explained proportion of information of the y-axis variable (normalised mutual information). This is an asymmetric measure, and magnitude does not represent directionality. We were only interested in the information between religion and specialty, so the rest of the data has been masked.

Supplementary Figure 3 shows Theil’s U for the GMC data. Knowing a doctor is a GP, gives us some amount of information on whether the GP’s faith would be Hindu or Sikh (light blue). Combined with the chi-squared analysis Figure 1B, the data informs us that knowing a doctor is a GP increases the chances that they would be Sikh and reduces chances that they would be Hindu (compared to not knowing anything about the doctor’s religious affiliation or specialty). We also see that some of the same correlations from HEE data carry through to entry on the GMC specialist register, such as the association of Muslims with anaesthetics. Note that Theil’s U can be sensitive to the prior distribution, such as low numbers in occupational medicine, contrary to the chi-squared analyses, and Theil’s U results should be interpreted in the context of Supplementary Figure 1, as below.


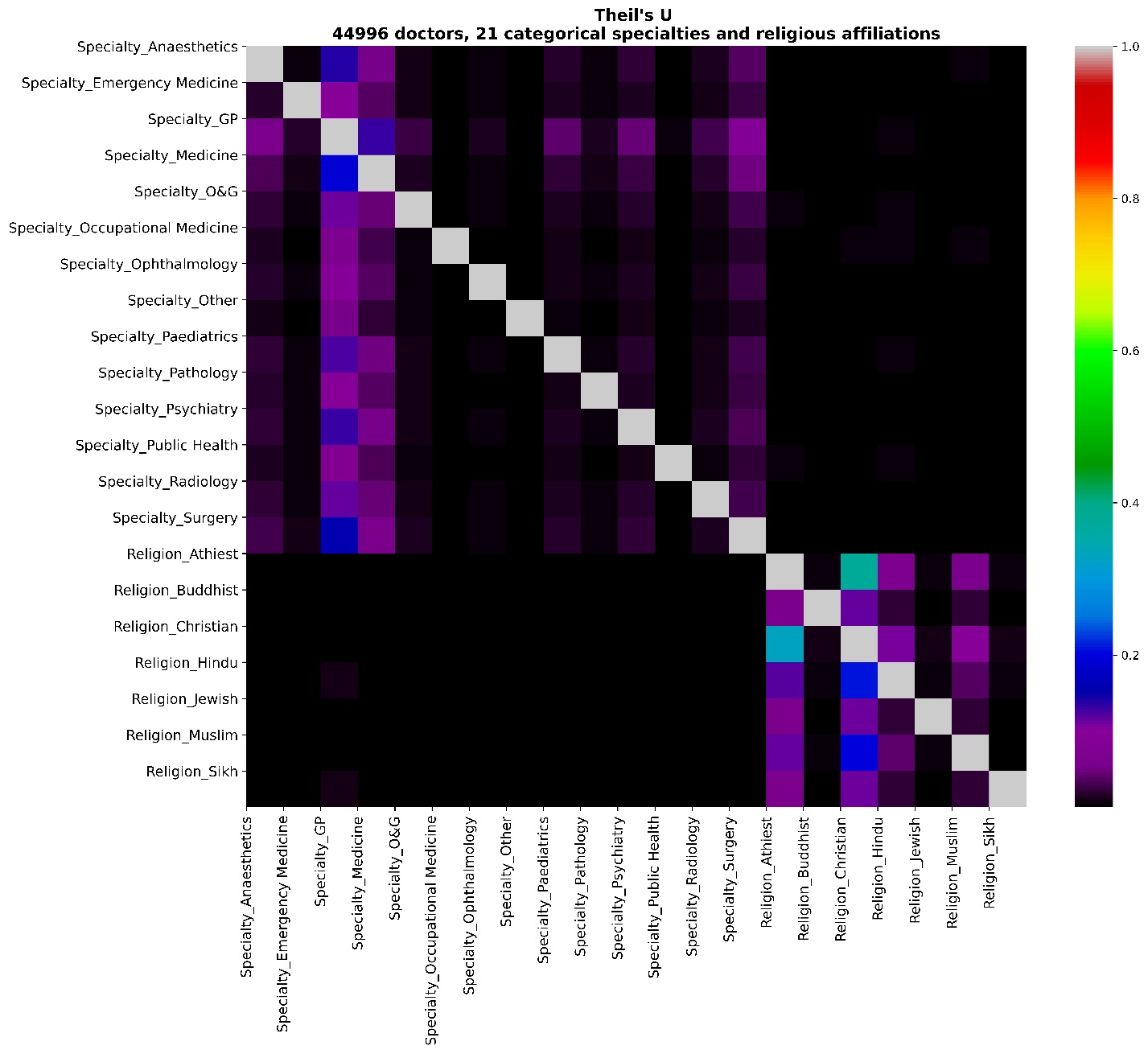


**Supplementary Figure 3**: Theil’s U (NMI) for all specialists on the register and their religious affiliations (GMC). Given knowledge of a variable on the x-axis, the colour scheme represents the explained proportion of information of the y-axis variable. This is an asymmetric measure, and magnitude does not represent directionality.

Supplementary Figure 2 and 3 exclude unknown and other religious affiliations, and are calculated from the same data as Tables 1 and 2 respectively (i.e., without data imputations).

### Results of Further Sensitivity Analyses

#### Multiple Tests of Proportions: Excluding and Including Unknown Religious Affiliations and with Imputation/Redistribution

The results for HEE applications to specialty data were relatively robust to inclusion or exclusion of the two unspecified religious affiliation categories (“other” and “unknown”) (Figure 1). The results for GMC data were not. This is not unexpected given the proportion of combined “other” + “unknowns” in the GMC data from a total of 143,081 specialists is 68.5% (Supplementary Tables 3 and 4).

#### Sensitivity to methods of Data Imputation: Proportional Redistribution of unknowns

It is not clear why there was such a discrepancy between the GMC specialist registrations, where the percentages of Christians and Atheists were significantly less than that of the general UK doctors’ population (Supplementary Table 4).

We redistributed the newly allocated numbers from the unknown group to each religion, based on the existing distribution of UK doctors by faith. The resulting chi-squared analyses, showing only the significant results at 0.5% alpha significance after Bonferroni correction, and normalised to expected frequencies, is show in Supplementary Figure 4 (E, F, G, H).

We further carried out the same data redistribution but using the England and Wales general population data (instead of the UK Doctors’ data). The England and Wales data from Supplementary Table 1 has the least unknown religious affiliations and so this was to assess sensitivity to the degree of redistribution of the unknown category, specifically for the case of the GMC data. These are shown in Supplementary Figure 4 (I, J, K ,L).

The results using different imputation methods mainly affected the GMC data, specifically atheist correlations but also Christian-GP and Sikh-GP correlations. Despite this, no sensitivity analysis effected the overall robustness of our results presented in the main manuscript – if we had also incorporated these imputation methods a priori, there would have been no overall change to the results.

| E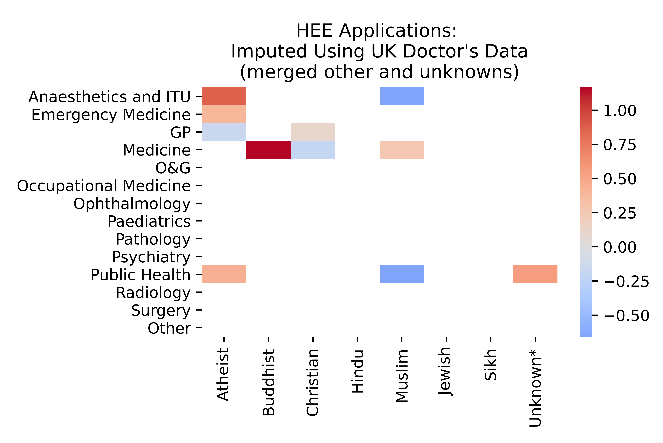 | F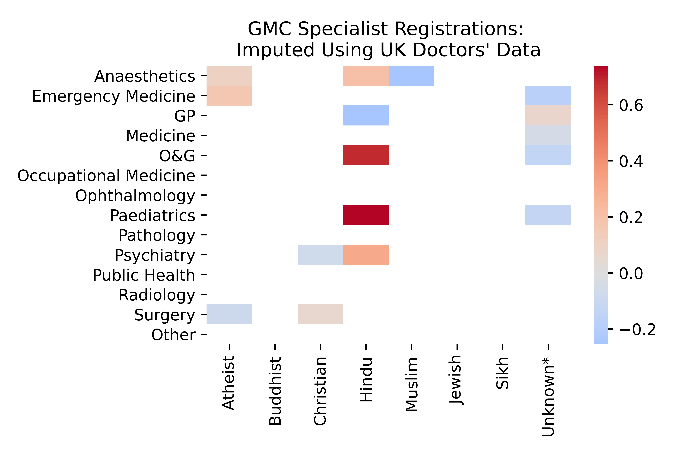 |
| --- | --- |
| G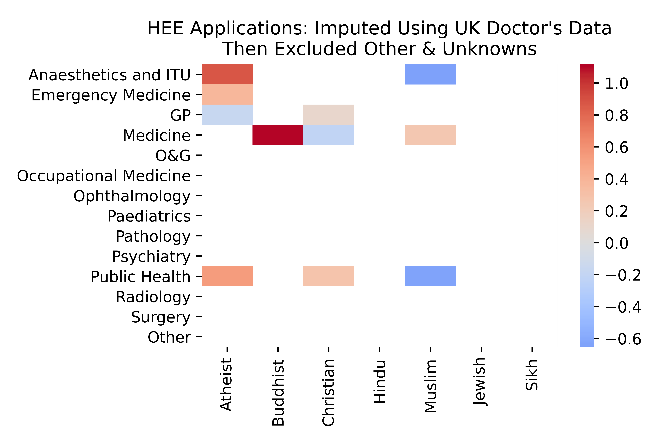 | H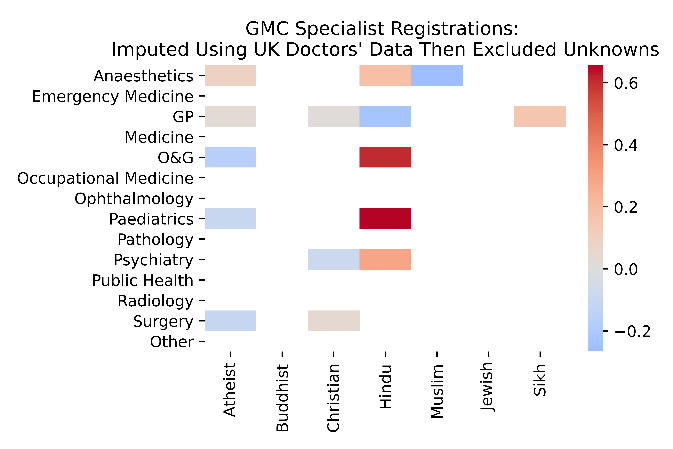 |
| I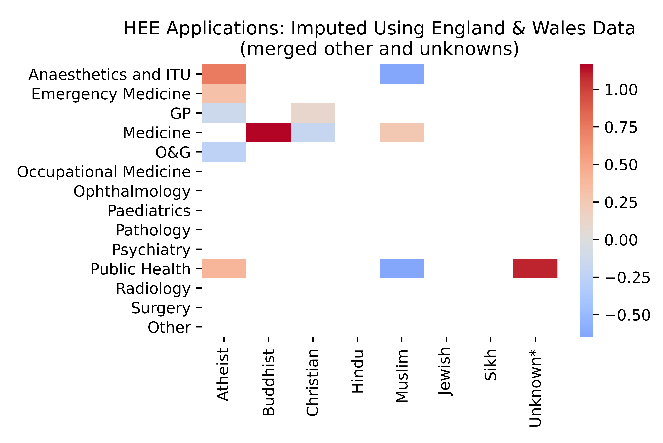 | J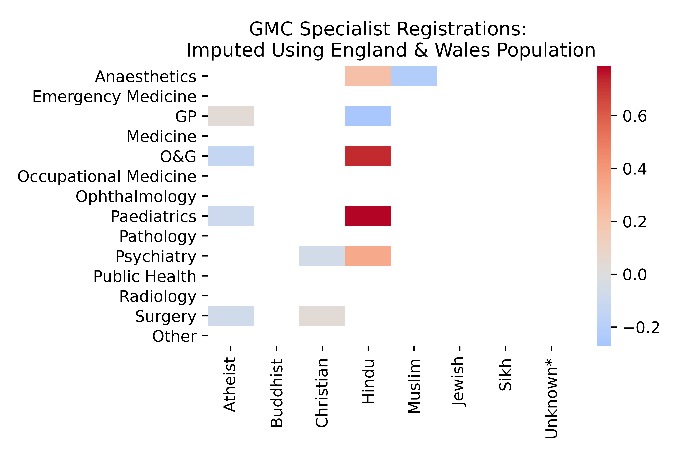 |
| K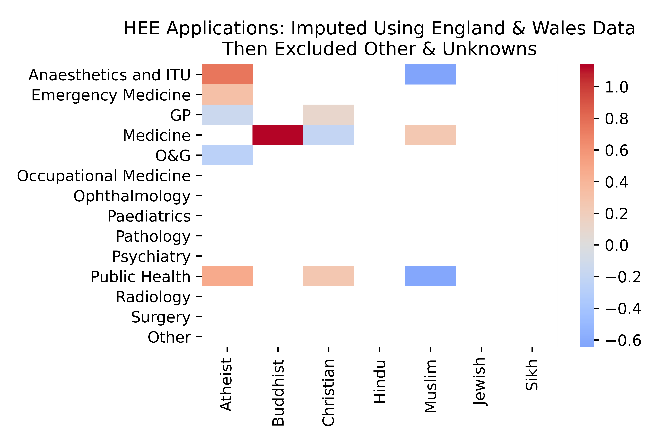 | L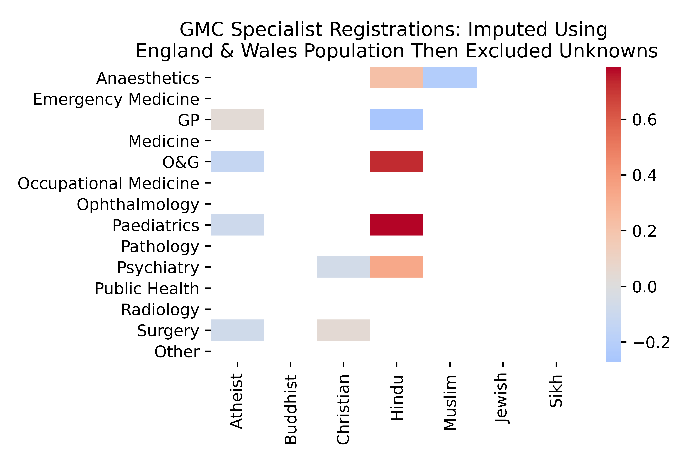 |

**Supplementary Figure 4:** GMC data imputed using UK Doctors’ data. As per Supplementary Figure 1F but magnified. This is after redistribution of unknowns using the general UK doctors’ data. These are active specialist registrations by faith (GMC 2019). Only the statistically significant two-by-two chi-squared results are shown (p<0.005 Bonferroni corrected). The data are normalised to the expected frequencies. *=combined other and unknown categories due to the external data from Table 4 used to redistribute the unknowns.

#### Sensitivity to prior values of alpha and Bonferroni correction

A change of alpha threshold from 0.005 to traditional alpha of 0.05 or even a reduction to 0.0005 only significantly affected one HEE data as it reduced the significance of Atheist applications to O&G.

Similarly, using all the various methods of imputation and interaction with Bonferroni correction for multiple comparisons did not affect the results except for the same atheist applications to O&G, as detailed in Supplementary Table 5 below.

Supplementary Figures 5 and 6 show the final robust overlays of the above analyses for the HEE and GMC data respectively.

### HEE Robust Result Overlays

The final results for specified religious affiliations are shown below. This keeps only the associations which were present in all sensitivity analyses and on NMI.


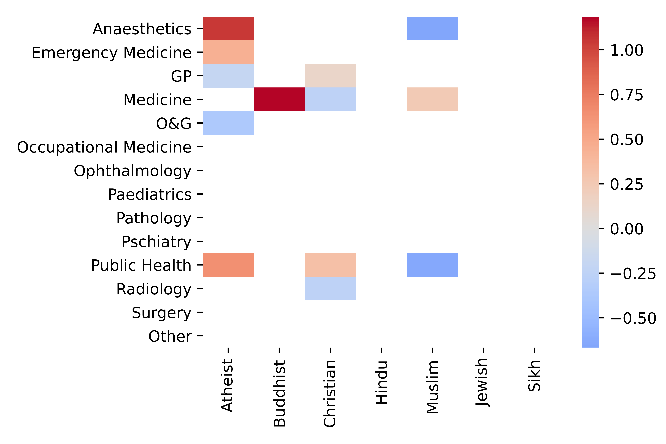


a, e, i, t

i

a, i

t

**Figure 5:** HEE Medical-Specialty/Religious-Affiliation correlations after sensitivity analyses. Only significant correlations that were robust in all sensitivity analyses are shown: multiple tests of proportions with and without unknown religious affiliations and imputed redistribution of unknown religious affiliations (p<0.005 Bonferroni corrected), and asymmetric NMI scores. The robust results are filled rectangles; white rectangles represent significant results in one or more tests. The colour bar is normalised to expected frequencies. a = correlation not present when all data included. t = not present in NMI scores. e = not present when unknown data excluded, i = not present on one or more data imputation methods.

| **Doctors of specified faith or no faith applying to specialty** | **“Relative Risk”**  **[99.5% CI]** | **Odds Ratio [99.5% CI]** | **Uncorrected p-value** | **Yates’ Chi-squared statistic** |
| --- | --- | --- | --- | --- |
| **Buddhist-Medicine** | 2.27 [1.91, 2.69] | 3.04 [2.33, 3.98] | 7.8 x10^‑34^ | 147.0 |
| **Muslim-Anaesthetics & ITU** | 0.27 [0.19, 0.38] | 0.26 [0.18, 0.37] | 1.2 x10^‑30^ | 132.4 |
| **Muslim-Medicine** | 1.4 [1.27, 1.54] | 1.51 [1.34, 1.71] | 1.2 x10^‑21^ | 91.3 |
| **Muslim-Public Health** | 0.27 [0.18, 0.39] | 0.26 [0.18, 0.38] | 2.1 x10^‑26^ | 113.1 |
| **Christian-Medicine** | 0.71 [0.64, 0.8] | 0.67 [0.59, 0.76] | 9.1 x10^‑18^ | 73.7 |
| **Atheist-Anaesthetics** | 2.51 [2.08, 3.04] | 2.68 [2.18, 3.29] | 5.0 x10^‑44^ | 193.7 |
| **Atheist-Emergency Medicine** | 1.64 [1.37, 1.96] | 1.71 [1.4, 2.08] | 1.2 x10^‑14^ | 59.5 |
| **Atheist-GP** | 0.79 [0.72, 0.86] | 0.70 [0.62, 0.79] | 1.1 x10^‑16^ | 68.7 |
| **Atheist-Public Health** | 1.67 [1.33, 2.09] | 1.71 [1.35, 2.18] | 2.1 x10^‑10^ | 40.4 |

Table 5.1: Relative Risks and Odds-Ratios for the robust **HEE** results. Calculated from two-by-two contingency matrices from Supplementary Table 1 with **unknown** data included. Magnitudes of results were generally robust to whether unknown data was included or not or chosen values for alpha and method of Bonferroni correction.

| **Doctors of specified faith or no faith applying to specialty** | **“Relative Risk”**  **[95% CI]** | **Odds Ratio [99.5% CI]** | **Uncorrected p-value** | **Yates’ Chi-squared statistic** |
| --- | --- | --- | --- | --- |
| **Buddhist-Medicine** | 2.25 [1.9, 2.67] | 3.02 [2.3, 3.95] | 5.0 x10^‑33^ | 143.3 |
| **Muslim-Anaesthetics & ITU** | 0.26 [0.19, 0.37] | 0.25 [0.18, 0.36] | 1.8 x10^-31^ | 136.2 |
| **Muslim-Medicine** | 1.41 [1.27, 1.56] | 1.52 [1.34, 1.73] | 7.9 x10^-21^ | 87.6 |
| **Muslim-Public Health** | 0.26 [0.18, 0.39] | 0.25 [0.17, 0.37] | 2.0 x10^-26^ | 113.2 |
| **Christian-Medicine** | 0.69 [0.61, 0.77] | 0.64 [0.56, 0.73] | 8.9 x 10^-21^ | 87.4 |
| **Atheist-Anaesthetics** | 2.74 [2.24, 3.35] | 2.93 [2.36, 3.64] | 1.2 x10^-47^ | 210.3 |
| **Atheist-Emergency Medicine** | 1.63 [1.36, 1.96] | 1.7 [1.39, 2.08] | 9.5 x10^-14^ | 55.5 |
| **Atheist-GP** | 0.77 [0.71, 0.84] | 0.68 [0.6, 0.77] | 1.3 x10^-18^ | 77.5 |
| **Atheist-Public Health** | 1.79 [1.41, 2.26] | 1.84 [1.44, 2.36] | 4.0 x10^-12^ | 48.2 |

*Table 5*.2*: Relative Risks and Odds-Ratios for the robust* ***HEE*** *results. Calculated from two-by-two contingency matrices from Supplementary Table 1* ***excluding*** *unknown data. Magnitudes of results were generally robust to whether unknown data was included or not. The RR column is greyed out because without the unknowns, the data is incomplete and RR should not be used, it is included here only for comparison to show minimal difference between including or excluding unknowns.*

### GMC Robust Result Overlays


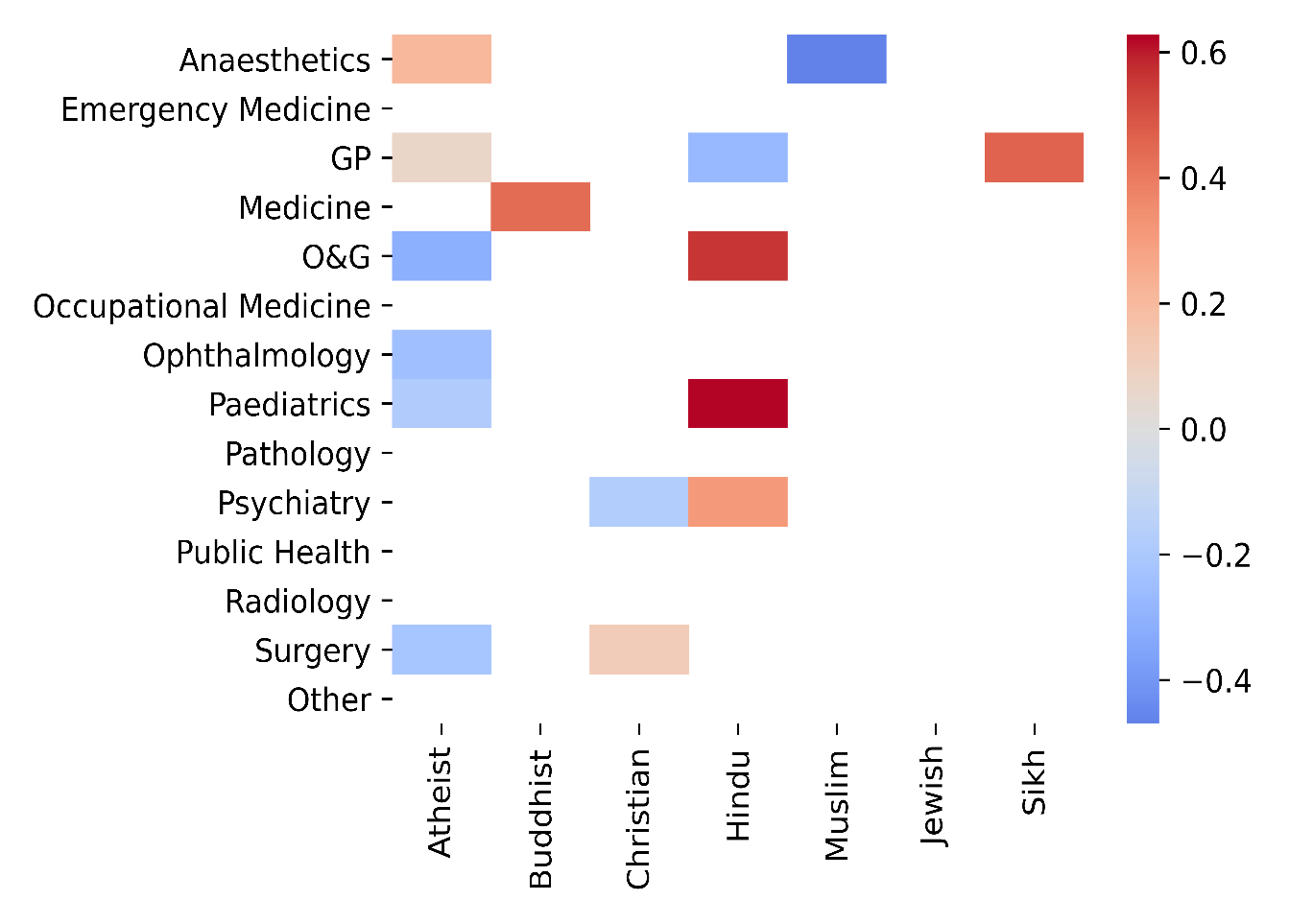


e, i, t

c(e, a), i, t

a

a, i, t

a, i, t

e, i, t

e, i, t

e, t

a, t

e, t

e, t

e, i, t

e, i, t

e, i, t

a

e, i, t

e, i

e, i, t

t, i

e, t

t

t

e, i, t

e, i, t

e, t

t

e, i, t

t, i

**Supplementary Figure 6:** GMC Medical-Specialty/Religious-Affiliation correlations after sensitivity analyses. Only significant correlations that were robust in all sensitivity analyses are shown: multiple tests of proportions with and without unknown religious affiliations (p<0.005 Bonferroni corrected), multiple tests of proportions after missing data imputation, and asymmetric NMI scores. The robust results are filled rectangles, otherwise white rectangles. The colour bar is normalised to expected frequencies. a = correlation not present when all data included. t = not present in NMI scores. e = not present when unknown data excluded. c = conflict in direction of correlation between two or more analyses, i = not present on data imputation methods.

| **Doctors of specified faith or no faith applying to specialty** | **“Relative Risk”**  **[99.5% CI]** | **Odds Ratio [99.5% CI]** | **Uncorrected p-value** | **Yates’ Chi-squared statistic** |
| --- | --- | --- | --- | --- |
| **Muslim-Anaesthetics & ITU** | 0.57 [0.47, 0.7] | 0.55 [0.45, 0.68] | 3.5 x10^‑16^ | 66.5 |
| **Hindu-GP** | 0.6 [0.56, 0.64] | 0.45 [0.41, 0.49] | 2.4 x10^-141^ | 640.6 |
| **Hindu-O&G** | 2.11 [1.79, 2.49] | 2.18 [1.83, 2.6] | 4.4 x10^-37^ | 161.9 |
| **Hindu-Paediatrics** | 2.2 [1.92, 2.51] | 2.31 [2.0, 2.67] | 1.0 x10^-62^ | 279.4 |

*Table 6.1: Relative Risks and Odds-Ratios for the robust* ***GMC*** *results. Calculated from two-by-two contingency matrices from Supplementary Table 2 with* ***unknown*** *data included. Magnitudes of results were generally robust to whether unknown data was included or not.*

| **Doctors of specified faith or no faith applying to specialty** | **“Relative Risk”**  **[99.5% CI]** | **Odds Ratio [99.5% CI]** | **Uncorrected p-value** | **Yates’ Chi-squared statistic** |
| --- | --- | --- | --- | --- |
| **Muslim-Anaesthetics & ITU** | 0.51 [0.41, 0.62] | 0.48 [0.39, 0.6] | 5.8 x10^-23^ | 97.3 |
| **Hindu-GP** | 0.71 [0.66, 0.76] | 0.6 [0.55, 0.66] | 2.7 x10^-54^ | 240.8 |
| **Hindu-O&G** | 1.69 [1.41, 2.01] | 1.73 [1.44, 2.08] | 8.5 x10^-17^ | 69.3 |
| **Hindu-Paediatrics** | 1.77 [1.52, 2.03] | 1.84 [1.58, 2.14] | 1.4 x10^-29^ | 127.5 |

*Table 6.2: Relative Risks and Odds-Ratios for the robust* ***GMC*** *results. Calculated from two-by-two contingency matrices from Supplementary Table 2 with unknown data* ***excluded****. Magnitudes of results were generally robust to whether unknown data was included or not.*

### How many training cohorts would it take to mitigate the deficit in Muslim consultant anaesthetists?

The minimum number of training cohorts for the Muslim-Anaesthetist deficit to be rectified is approximated by:

**Min cohorts =** $\frac{\boldsymbol{Expected no. of Muslim Consultant Anaesthetists - Actual no. of Muslim Consultant Aneasthetists}}{\boldsymbol{no. of Muslim applicants in 2019 (HEE) \times factor \times application success rate}}$

Where *factor* is the proportion by which Muslim applications to anaesthesia increase above that of 2019. The numerator is given by the GMC data and its expected chi-squared frequencies.

In the 2019 HEE data there were 70 Muslim junior doctor applicants to anaesthesia (Table 1). If the number of applications were to double compared to 2019, then factor is set to 2. In the 2020 GMC data, there were 197 Muslim consultant anaesthetists.

This simplifies to:

**Number of training cohorts required =** $\frac{\boldsymbol{Expected Muslims Consultant Anaesthetists - 197}}{\boldsymbol{70 \times2 \times application success rate}}$

**Including unknown GMC data**

The expected two-by-two frequencies for consultant anaesthetists is taken from the chi-squared test from the repository, specifically the notebook named “GMC_HEE_RR_OR_CI” (<https://github.com/thenineteen/GMC_HEE_2019/blob/master/GMC_HEE_RR_OR_CI.ipynb>)

This was used to make Table 6.1.

There were 340 expected Muslim consultant anaesthetists.

If success rate is 1/5 applicants getting into anaesthesia, then minimum no. of training cohorts required is 5 years. If success rate is taken to be 50%, this becomes 2 years.

**Excluding unknown GMC data**

Excluding unknowns, there were 370 expected Muslim consultant anaesthetists.

If success rate is 1/5 applicants getting into anaesthesia, then minimum no. of training cohorts required is 6 years. If success rate is taken to be 50%, this becomes ~ 2½ years.

Therefore, the number of applications by Muslim doctors to A&IC needs to more than double (compared to applications in 2019) for two years for this deficit to be mitigated over two training cohorts, assuming an optimistic 50% application success rate (Appendix).

### Are foreign trained Muslim doctors equally likely to be Consultant Anaesthetists?

To explore other confounding variables that might explain the association between Muslim doctors and Anaesthetics specialty, we conducted sub-group analysis using ethnicity and whether doctors were foreign trained. This analysis was only conducted for the GMC data as this information was not available from HEE.

For the sub-group analysis, we have focused on the association between Muslim doctors and Anaesthetics specialty as this was the strongest association found from the post-hoc tests.

When the data was split according to whether doctors were foreign trained (IMG PMQ) or UK train (UK PMQ) we found a significant association between religion and specialty (for UK trained: p-value=8.36e-135, foreign trained: p-value=2.64e-98). The relative risk for the association between Muslim religion and Anaesthetic specialty in the sub-groups showed a lot of overlap which implied that the association found earlier was irrespective of the Muslim doctors’ primary qualification (UK vs foreign).

| **Statistics** | **UK PMQ & Muslim** | **IMG PMQ & Muslim** |
| --- | --- | --- |
| **Chi-square (p-value)** | 8.36e-135 | 2.64e-98 |
| **RR (Muslim - A&IC)** | 0.54 (0.44, 0.66) | 0.56 (0.49, 0.63) |
| **Entire Muslim data** | 0.57 [0.47, 0.7] | |

Table 6.3: Sub-group analysis results when GMC data was split according to whether doctors had a UK primary qualification (UK PMQ) or a foreign primary qualification (IMG PMQ). The Chi-square p-value is for the overall association between religion and specialty.

The sub-group analysis for ethnicity was conducted to check if the association between Muslim religion and Anaesthetics specialty was stronger in some ethnic groups than others. We focused on those ethnic groups whose proportion of Muslims was above the England and Wales benchmark of 5.7%. The result showed a strong significant association between Muslim religion and Anaesthetic specialty among the Asian ethnic nationalities. This was also the ethnic group with the largest population of Muslims.

| **Statistics\Ethnicity** | **Asian** | **Black** | **Mixed** | **Other** |
| --- | --- | --- | --- | --- |
| **n** | 6094 | 544 | 268 | 1391 |
| **RR** | 0.48 | 0.81 | 0.93 | 1.24 |
| **CI** | (0.42, 0.55) | (0.50, 1.33) | (0.59, 1.47) | (0.98, 1.58) |
| **p-value** | 4.46e-31 | 0.47 | 0.90 | 0.07 |
| **Entire Muslim data** | 0.57 [0.47, 0.7] | | | |

Table 6.4: Relative risk for the association between Muslim religion and Anaesthetic specialty by ethnic group.

To understand if Asian ethnicity was the only reason for the association found between Muslim religion and Anaesthetic specialty, we computed the association between Asian ethnic group and Anaesthetic specialty (RR=0.84, CI: (0.81, 0.88), p-value= 2.06e-17). This showed that without accounting for Muslim religion, the relative risk was 0.84 (CI: (0.81, 0.88)) and after adjustment for Muslim religion, the relative risk become 0.48 (0.42, 0.55). This supports our earlier claim of Muslim doctors being less likely to be in Anaesthetic specialty compared to doctors of other religious affiliation.

## Summary and Conclusions

The summary results from all tests are summarised in Fig. 7.

We looked at the current state of specialists in the UK by faith (GMC data), and the future (HEE applications to specialties in 2019 data). We performed statistical tests to determine whether doctors of any faiths had a predisposition towards particular specialties. Due to a low prior, we chose a reduced statistical significance threshold of 0.005 and performed conservative global Bonferroni corrections for multiple comparisons. Due to a significant proportion of unknown faiths in the GMC data, we performed sensitivity analyses. We only kept the associations that were statistically significant on all four tests for each dataset (3 tests of proportions and 1 asymmetric normalised mutual information score) and reported these. Furthermore, we investigated how robust our faith-specialty preferences were by altering the method of data imputation and whether or not doctors with an undeclared religious affiliation were included in the tests of proportions using a further 3 tests per dataset (in total four tests investigating sensitivity to imputation method for each dataset).

The most significant trend was that currently, there are disproportionately fewer Muslim anaesthetists and judging by the applications in 2019 where Muslims were 73% less likely to apply for anaesthetics, this trend is set to continue (RR 0.27 95% CI [0.21, 0.34], p=1.2 x10^‑30^). This was the only association present on both datasets. Applications by Muslims to Anaesthesia need to at least double for two years for the current deficit to be mitigated in the future (the expected number of Muslim applications to anaesthetics was 214, vs the actual 70; the expected number of Muslims anaesthetists on the GMC register was 340, the actual number was 197).

The current snapshot also shows that there are disproportionately higher numbers of Hindu O&G and Paediatric consultants on the GMC register, however, these associations were not found on applications by Hindu doctors in 2019, and so may dwindle with time.

A potential glimpse at the future of the religious makeup of the specialist workforce by analysing the HEE 2019 data shows unexpected associations: atheist doctors were two-and-a-half times more likely to apply to Anaesthesia than doctors of other faiths (RR 2.51 95% CI [2.2, 2.87], p=5.0 x10^‑44^), atheists also preferred to apply to Emergency Medicine and Public Health while they were 21% and 36% less interested in GP and O&G respectively. More than twice as many Buddhists apply to Medical specialties than expected (normalised ratio above 1.0) and also twice as likely to apply to Medicine than any other faith group (OR=3.0 95% CI [2.5, 3.7], RR=2.30 95% CI [2.0, 2.6], p=0.0, Yates’ chi-square=144.99, dof=1). Muslims were 73% less likely to apply to public health (RR 0.27 95% CI [0.21, 0.35], p=2.1 x10^‑26^).

These results were all very robust.


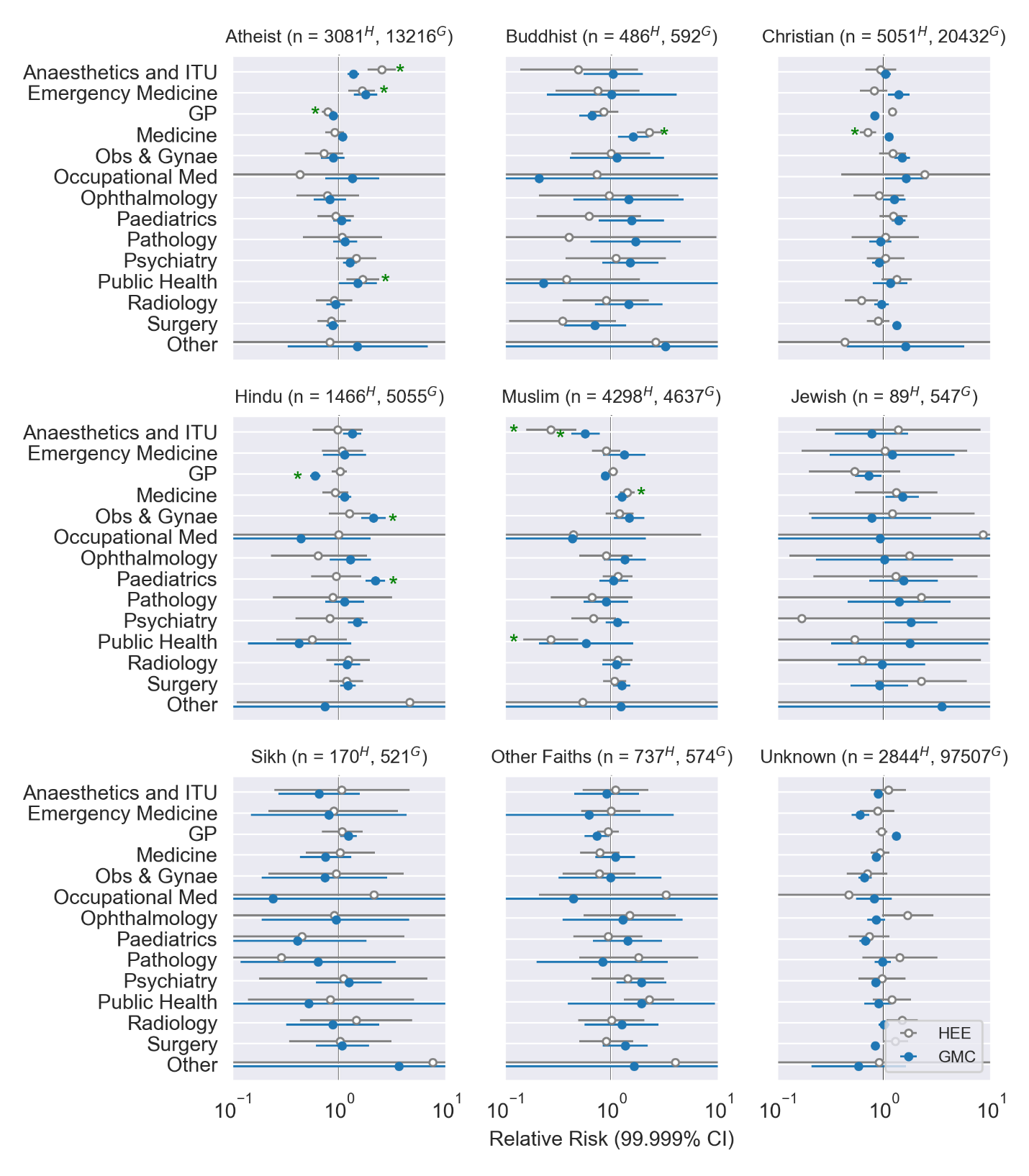


Figure 7: summary HEE and GMC relative risks. At the reduced alpha of 0.5% after Bonferroni correction, this leaves us with 99.999% confidence intervals. Green stars signify that the association persisted across all 7 sensitivity analyses. Fewer Muslim anaesthetist and ITU applicants in 2019 and consultants in 2020 were the only associations that persisted for both datasets.

## Supplementary References

1. Virtanen P, Gommers R, Oliphant TE, et al. SciPy 1.0: fundamental algorithms for scientific computing in Python. *Nature methods.* 2020:1-12.

2. Camerer CF, Dreber A, Holzmeister F, et al. Evaluating the replicability of social science experiments in Nature and Science between 2010 and 2015. *Nature Human Behaviour.* 2018;2(9):637-644.

3. Benjamin DJ, Berger JO, Johannesson M, et al. Redefine statistical significance. *Nature Human Behaviour.* 2018;2(1):6-10.

4. Wikipedia. Uncertainty coefficient. In Wikipedia, The Free Encyclopedia Web site. <https://en.wikipedia.org/w/index.php?title=Uncertainty_coefficient&oldid=850346922>. Published 2018, July 15. Accessed July 2, 2019.

5. Religion, education and work in England and Wales. Office for National Statistics. <https://www.ons.gov.uk/peoplepopulationandcommunity/culturalidentity/religion/datasets/religioneducationandworkinenglandandwales>. Published 2020. Accessed 15/04, 2020.

6. UK workforce demographics NHS. <https://digital.nhs.uk/about-nhs-digital/corporate-information-and-documents/how-we-support-diversity-and-inclusion/our-workforce-demographics-2019/religion>. Published 2019. Accessed 15/04, 2020.
